# An alkaloid initiates phosphodiesterase 3A–schlafen 12 dependent apoptosis without affecting the phosphodiesterase activity

**DOI:** 10.1038/s41467-020-17052-4

**Published:** 2020-06-26

**Authors:** Youwei Ai, Haibing He, Peihao Chen, Bo Yan, Wenbin Zhang, Zhangcheng Ding, Dianrong Li, Jie Chen, Yan Ma, Yang Cao, Jie Zhu, Jiaojiao Li, Jinjie Ou, Shan Du, Xiaodong Wang, Jianzhang Ma, Shuanhu Gao, Xiangbing Qi

**Affiliations:** 1 College of Wildlife and Protected Area, Northeast Forestry University, Hexing Road, 150040 Harbin, China; 2 National Institute of Biological Sciences, 7 Science Park Road, Zhongguancun Life Science Park, 102206 Beijing, China; 3 Tsinghua Institute of Multidisciplinary Biomedical Research, Tsinghua University, Beijing, China; 4 Shanghai Engineering Research Center of Molecular Therapeutics and New Drug Development, East China Normal University, 3663N Zhongshan Road, 200062 Shanghai, China

**Keywords:** Apoptosis, Target identification

## Abstract

The promotion of apoptosis in tumor cells is a popular strategy for developing anti-cancer drugs. Here, we demonstrate that the plant indole alkaloid natural product nauclefine induces apoptosis of diverse cancer cells via a PDE3A-SLFN12 dependent death pathway. Nauclefine binds PDE3A but does not inhibit the PDE3A’s phosphodiesterase activity, thus representing a previously unknown type of PDE3A modulator that can initiate apoptosis without affecting PDE3A’s canonical function. We demonstrate that PDE3A’s H840, Q975, Q1001, and F1004 residues—as well as I105 in SLFN12—are essential for nauclefine-induced PDE3A-SLFN12 interaction and cell death. Extending these molecular insights, we show in vivo that nauclefine inhibits tumor xenograft growth, doing so in a PDE3A- and SLFN12-dependent manner. Thus, beyond demonstrating potent cytotoxic effects of an alkaloid natural product, our study illustrates a potentially side-effect-reducing strategy for targeting PDE3A for anti-cancer therapeutics without affecting its phosphodiesterase activity.

## Abstract (web summary)

PDE3A modulators for cancer therapy cause serious side effects as they inhibit PDE3A phosphodiesterase activity, which is essential for the maturation of oocytes and the formation of platelets. Here, the authors identify a compound, nauclefine, that does not inhibit PDE3A activity but induces apoptosis by enabling a complex formation between PDE3A and SLFN12.

## Introduction

During the 1980s, researchers purified cAMP phosphodiesterase (PDE) in human platelets and in bovine hearts and discovered that its activity could be inhibited by the cGMP and PDE inhibitor compound milrinone^1,2^. This cGMP-mediated inhibition of cAMP PDE activity was later shown to be mediated by PDE3 family proteins (e.g., PDE3A and PDE3B)^1,3,4^. A breakthrough study in 1992 revealed the full-length cDNA sequence of *PDE3A*^4^, and extensive subsequent work has revealed that PDE3A occurs as at least three isoforms in cells, each with distinct N-terminal regulator domains but identical C-terminal phosphodiesterase domains^1^. The affinity of PDE3A for cGMP is higher than cAMP, but the *V*_max_ of PDE3A for cAMP is tenfold higher than for cGMP, explaining the observation that cGMP can readily inhibit the PDE3A-mediated hydrolysis of cAMP^5^. Biochemical experiments with truncation variants established that the PDE3A C-terminal domain alone is sufficient for the hydrolysis of its second message substrates cAMP and cGMP^6^. PDE3A’s regulation of cellular cAMP and cGMP concentrations has been shown to control biological processes including maturation of oocytes, contraction of muscles, and vasodilation^7,8^. Inhibition of the PDE activity of PDE3A by inhibitors can affect the maturation of oocytes, decrease the formation of platelets, and increase the risk of sudden death in patients with cardiac diseases^7–10^.

Beyond these long-studied second-messenger-mediated regulatory functions of PDE3A, an increasing number of studies have reported cAMP- and cGMP-independent functions of PDE3A in promoting cancer cell apoptosis in recent years^11–23^. Some PDE3 PDE enzyme inhibitors were found to be cytotoxic to cancer cells^11^, and later work which combined chemogenomic analysis revealed that the cytotoxic PDE3 inhibitor DNMDP promotes apoptosis of HeLa cells through its binding with PDE3A^13,21,22^.

Later, work in basic oncology has revealed that PDE3 inhibitors including zardaverine, quazinone, and anagrelide can induce apoptosis in tumor cell types that feature PDE3A expression (e.g., HeLa, H4, MCF7, EKVX, A2058, SK-MEL-3, HCC, ERMS, A2058, COLO741, H2122, and GIST882 cells)^11–13,15,17–19,21–23^. Importantly, these studies showed that the inhibitors kill cells independent of the canonical cAMP and cGMP hydrolysis activity of PDE3A^12,13,19,21^ and, moreover, established that the killing can be blocked by co-treatment with noncytotoxic PDE3 inhibitors including cilostazol and trequinsin^13,19^. Recently, our lab demonstrated that estrogen and related steroids can induce a PDE3A-dependent form of cell death that is likewise independent of PDE3A’s enzymatic activity^19^. These findings have motivated extensive research to target PDE3A in pursuit of effective anticancer therapies. However, the present cytotoxic PDE3A agents also inhibit the PDE activity of PDE3. Therefore, developing PDE3A modulators which can initiate PDE3A–SLFN12-dependent cell death but do not affect the PDE activity is currently urgent.

Natural products represent a huge source of diverse new drugs, among which the more than 4000 known indole alkaloids represent one of the largest and most important chemical classes due to their potent and highly varied physiological activities. Extracts and compounds isolated from different parts of *Nauclea latifolia* plants, especially indoloquinolizidine alkaloids, also exhibit a broad spectrum of bioactivities^24–26^. Herein, we report that the indole alkaloid natural product nauclefine^27,28^—initially characterized from the bark of *Nauclea subdita*—can induce PDE3A–SLFN12-dependent apoptosis of HeLa cells. Biochemically, pull-down experiments revealed that nauclefine binds PDE3A. Unlike other known PDE3A modulators, nauclefine does not inhibit the PDE activity of PDE3A, representing a type of PDE3A modulator that induces cancer cell death without affecting its canonical function.

## Results

### The indole alkaloid nauclefine induces apoptosis of cells

**Fig. 1:**
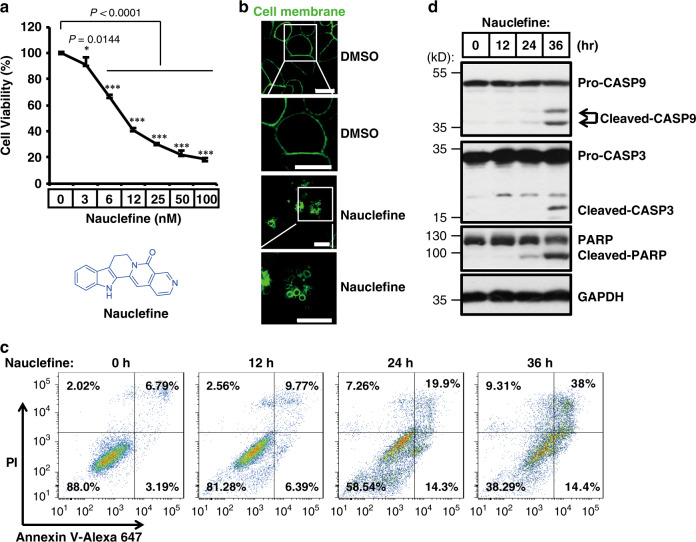
Nauclefine induces apoptosis of HeLa cells. **a** HeLa cells were treated with the indicated concentrations of nauclefine for 48 h, then cell viability was determined by measuring the ATP level (*n* = 4). The chemical structure of nauclefine is shown. Representative data are shown from three independent experiments (mean ± SD) and the *p* values from two-tailed unpaired Student’s *t* tests are indicated. *p < 0.05, **p < 0.01, ***p < 0.001. **b** HeLa cells were transfected with the phospholipase C Delta 1-GFP (PLCD1-GFP) vector, then cells were treated with DMSO or nauclefine (200 nM). Pictures were taken under confocal microscopy. Experiments were repeated twice. Scale bars, 10 µM. **c** HeLa cells were treated with nauclefine (200 nM) for the indicated times, then cells were stained for PI and Annexin V, and analyzed by flow cytometry. **d** HeLa cells were treated with 200 nM nauclefine for the indicated times, then cells were collected for western blot analysis. Western blots represent three independent replicates.

Our group previously reported a flexible strategy for the construction of indolizinone and quinolizinone scaffolds based on a cascade exo hydroamination reaction followed by spontaneous lactamization; this was used to synthesize camptothecin and several analogs^29^. To explore the potential application of these indoloquinolizidine-type molecules, we tested the cytotoxicity of these compounds against several cancer cell lines (Supplementary Fig. 1a). We were excited to observe that one type of indolizinone scaffold could kill cancer cells (assayed by Cell Titer-GLO based on ATP levels to indicate cell viability at 48 h post-treatment) (Supplementary Fig. 1a). Subsequently, structure–activity relationship studies were conducted (Supplementary Note). To our delight, we identified that one of the Nauclea alkaloids, nauclefine, could kill HeLa cells with an IC_50_ < 10 nM (Fig. 1a and Supplementary Fig. 1a). Further structure modification was then applied by varying the substitution group on the core structure, surprisingly, we found that a very similar compound, 7 (with only one atom difference, N vs. C), elicited no obvious phenotype, even at a 10 µM concentration (Supplementary Fig. 1b). Further varying the pyridine ring resulted in diverse nauclefine analogs, however, the activities of these modified nauclefines were either lower or diminished (Supplementary Fig. 1b). Our results indicated a role for the unique pyridine motif in nauclefine’s cytotoxic function. As only nauclefine (**1**) exerted the dramatic cell death-inducing effect (Supplementary Fig. 1), we next focused on the biochemical actions of this molecule.

Confocal microscopy revealed that nauclefine caused formation of typical apoptotic bodies in treated but not control cells with GFP-marked plasma membranes (Fig. 1b). During apoptosis, phosphatidylserine—which normally resides in the inner leaflet of the plasma membrane—is exposed on the external leaflet and can be stained by Annexin V-FITC^30^. As apoptosis progresses, membrane permeability increases and apoptotic cells can be stained using PI. We found that nauclefine caused a time-dependent increase in the intensities for Annexin V and PI stains (Fig. 1c and Supplementary Fig. 2). Further, caspase-9/3 were both activated and the caspase3 substrate PARP was cleaved upon nauclefine treatment (Fig. 1d), collectively indicating that nauclefine can cause apoptosis.

### PDE3A and SLFN12 are required for nauclefine-induced death

**Fig. 2:**
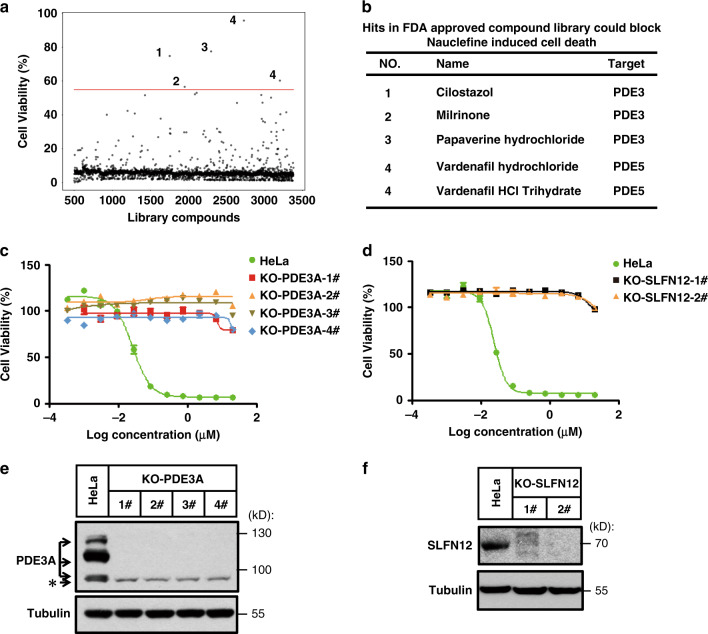
Nauclefine-induced cell death requires expression of PDE3A and SLFN12 in HeLa cells. **a** Primary screen results of cell viability protection by compounds of the FDA-approved library. Compounds 1–4 were potential hits that potently protect against nauclefine-induced cell death. **b** The name and previously known targets of FDA-approved compounds 1–4. Indicated concentrations of nauclefine used to treat HeLa cells with PDE3A knocked out (**c**) or SLFN12 knocked out (**d**) for 48 h. ATP levels were measured to assess cell viability. Data shown are from two independent experiments (with *n* = 4 wells each time). Expression of PDE3A (**e**) or SLFN12 (**f**) was knocked out using the CRISPR-Cas9 system, and was verified by western blotting. The western blot presents representative results from two experiments.

To identify the molecular mechanisms underlining nauclefine-induced cell death, we screened an FDA-approved library comprising about 3000 compounds (Supplementary Fig. 3a). Given that the inhibition targets of the individual drugs in the library are already known, any hit compounds which can block nauclefine-induced cell death would implicate the potential involvement of its corresponding target in this death pathway. The screen identified four hits that potently blocked nauclefine-induced cell death (Fig. 2a). Conspicuously, all of the annotated targets for these four hits were PDE, with one reported as a nonspecific PDE inhibitor, two PDE3 inhibitors, and one PDE5 inhibitor with different salt forms (Fig. 2b). We used titration to further characterize the abilities of these molecules to block nauclefine-induced cell death: all hits repeatedly blocked nauclefine-induced cell death, and did so in a dose-dependent manner (Supplementary Fig. 3b–d). Note that PDE5 expression in HeLa cells is negligible^11^, so we speculate that the ability of these PDE inhibitors to block nauclefine-induced cell death most likely results from targeting PDE3.

The Meyerson group and our own lab independently discovered that both DNMDP and estrogen (E2) can each induce a form of cell death that can be blocked by PDE3 inhibitors (including cilostazol and trequinsin), and identified that PDE3A is functionally involved in these processes^13,19^. We found in the present study that nauclefine-induced cell death can be blocked by cilostazol (Supplementary Fig. 3b) and trequinsin (Supplementary Fig. 3e), and these findings prompted us to examine potential functional roles for PDE3A in nauclefine-induced cell death. In addition, we also tested the involvement of SLFN12—a binding partner of PDE3A^13^ —in this death process. After confirming the lack of PDE3A and SLFN12 in generated CRISPR-Cas9-editing based PDE3A and SLFN12 knockout mutants (Fig. 2e, f), we conducted titration experiments with nauclefine and found that nauclefine-induced cell death was totally blocked in both knockout cell lines (Fig. 2c, d). These results establish that nauclefine-induced cell death discovered here is a PDE3A- and SLFN12-dependent form of cell death, as with the previously reported PDE3A modulators DNMDP and E2 (Supplementary Fig. 4). On the other hand, although those structurally unrelated cytotoxic compounds can induce PDE3A–SLFN12-dependent cell death, we conducted experiments and found that many clinically used anticancer agents induce cell death in a manner that is unrelated to PDE3A- and SLFN12-dependent form of cell death (Supplementary Fig. 5). Thus, PDE3A–SLFN12 axis clearly has divergent selectivity toward multiple cytotoxic agents.

Given the occurrence of at least three natural isoforms of PDE3A in HeLa cells^19^, we generated constructs for three isoforms: each having a different N-terminal regulatory domain but sharing an identical C-terminal PDE domain. Lentiviral expression of the three isoforms in PDE3A-KO HeLa cells rescued sensitivity to nauclefine (Supplementary Fig. 6a, b), indicating that all three isoforms are capable of mediating nauclefine-induced cell death. We also expressed SLFN12 in SLFN12-KO HeLa cells and found that reintroduction of SLFN12 rescued nauclefine-induced cell death (Supplementary Fig. 6c, d). Further, guided by a previous study reporting that a single amino acid mutation (I135N) in mouse SLFN2 causes myeloid and lymphoid immunodeficiency^31^, we mutated the corresponding amino acid in human (I105) to N in SLFN12 and found that this I105N mutation abolished SLFN12’s function in mediating nauclefine-induced cell death (Supplementary Fig. 6c, d).

### Nauclefine binds PDE3A but does not inhibit its PDE activity

Small molecules with a variety of structures (e.g., cAMP, cGMP, cilostazol, trequinsin, and other PDE3 inhibitors) can bind PDE3A, revealing that PDE3A has an apparently diverse binding capacity. Based on a DNMDP-2L small molecule probe, the Meyerson group found that DNMDP can bind PDE3A to induce PDE3A–SLFN12 interaction^13,22^. Our research group previously reported that estradiol (E2) can directly bind PDE3A based on an experimental strategy that combined pulldown and LC–MS/MS analysis^19^. We here used the well-established pulldown-then-LC–MS/MS method to assess whether nauclefine can bind PDE3A to initiate PDE3A- and SLFN12-dependent death (Supplementary Fig. 7a). Given that the C-terminal enzyme domain is functionally identical for cell death induction with full-length PDE3A^22^, and considering that both DNMDP and E2 were reported to bind the C-terminal domain of PDE3A^19,22^, we decided to use the C-terminal domain of PDE3A in this assay. Briefly, we transfected 293T cells with empty vector, Myc-tagged wild-type (WT) PDE3A (aa 669–1141) variant, and variants bearing amino acid mutations including H840A, Q975A, Q1001A, and F1004A (variants previously reported to affect PDE activity of PDE3A)^6,32^. Note that the Q1001 residue of PDE3A was previously reported to form H-bonds with cAMP and F1004 residue was reported to form van der Waals contacts with cAMP^33,34^. Then, we treated cells with nauclefine before lysed cells and performed anti-Myc immunoprecipitation to precipitate PDE3A variant proteins and the potential binding nauclefine (Supplementary Fig. 7a).

**Fig. 3:**
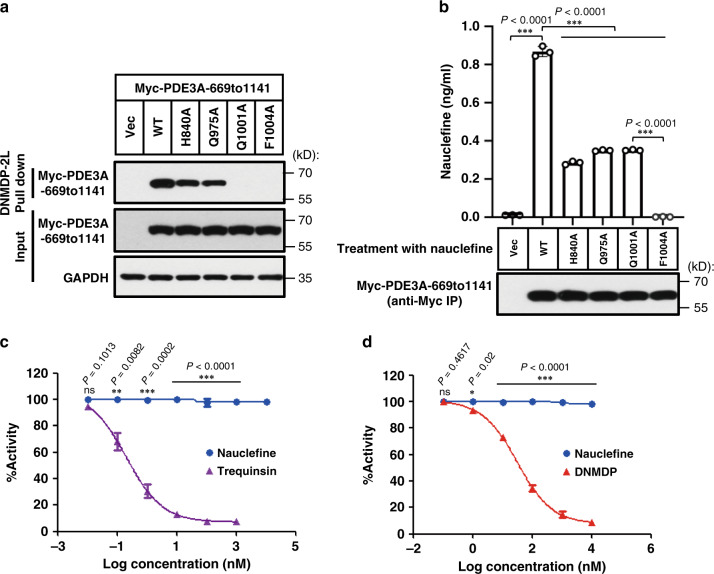
Nauclefine binds PDE3A without affecting its phosphodiesterase activity. **a** Pull-down assay of DNMDP-2L binding to Myc-tagged PDE3A (aa 669–1141) WT and to the indicated mutant variants. This is a representative result from three independent experiments. **b** Myc-tagged PDE3A (aa 669 to 1141) WT and the indicated mutant variants were immunoprecipitated with Myc-beads. The bound PDE3A proteins were detected by western blotting. The bound nauclefine was quantified via LC–MS/MS. The presented result (mean ± SD) is representative of two experiments with three replications in each experiment. Student’s *t* test (two-tailed, unpaired) was performed. The indicated concentrations of trequinsin and nauclefine (**c**), or DNMDP and nauclefine (**d**) were added to the insect-cell purified PDE3A protein in the presence of cAMP for 90 mins. At the end of the assay, the remaining cAMP was measured by LC–MS/MS. This is a representative result (mean ± SD) from two independent experiments with three replicates each time. Student’s *t* test (two-tailed, unpaired) was performed: *p < 0.05; **p < 0.01; ***p < 0.001; ns not significant.

We initially confirmed the well-established binding of these PDE3A variants with DNMDP-2L^13,22^: after pulldown, we confirmed that nauclefine binds WT PDE3A (Fig. 3a). The H840A and Q975A variants decreased the extent of PDE3A–DNMDP binding (Fig. 3a). The Q1001A and F1004A mutations totally blocked PDE3A–DNMDP binding (Fig. 3a). Based on a pulldown-then-LC–MS/MS strategy we found that nauclefine binds to WT PDE3A variant protein (Fig. 3b). The binding of nauclefine with WT PDE3A variant protein could be out-competed by the previously known noncytotoxic PDE3A inhibitors cilostazol and trequinsin (Supplementary Fig. 7b, c). We immunoprecipitated a similar amount of Myc-PDE3A proteins (WT and mutant variant groups) and found the H840A and Q975A mutations also significantly reduced nauclefine–PDE3A binding, and the F1004A mutation totally abolished nauclefine–PDE3A binding (Fig. 3b). However, we found that the Q1001A mutation only partially blocked nauclefine–PDE3A binding (Fig. 3b), a contrasting result compared with the impact of Q1001A in totally abolishing DNMDP–PDE3A binding (Fig. 3a); this distinction reveals a separate binding mode for the nauclefine–PDE3A and DNMDP–PDE3A interactions. We remain cautious: although we now have evidence indicating how the mutation of particular amino acids affects nauclefine binding with PDE3A, we do not firmly assert that the these selected amino acids directly interact with nauclefine; perhaps these residues actually function in proper active pocket conformation in a way that alters nauclefine’s binding with PDE3A indirectly.

Although the specific contribution(s) of these amino acids to small molecule binding is not fully understood, our results unequivocally demonstrate that nauclefine binds PDE3A using a binding model that is distinct from DNMDP–PDE3A binding. As DNMDP bound PDE3A and inhibited the PDE activity of PDE3A, we investigated whether nauclefine–PDE3A binding could affect the PDE activity of PDE3A. We used the well-known PDE3 inhibitors trequinsin and DNMDP as positive controls, and confirmed that both small molecules dose-dependently inhibited the PDE activity of PDE3A (Fig. 3c, d); in contrast, nauclefine did not affect the PDE activity of PDE3A, even when given at a concentration 1000 times higher than its IC_50_ concentration in cells (Fig. 3c, d). These striking results establish that although nauclefine definitely does bind to PDE3A, nauclefine does not inhibit PDE3A’s PDE activity, suggesting it is a new type of PDE3A modulator. Accordingly, nauclefine may enable targeting PDE3A without causing some of the PDE activity-related side effects that have limited the clinical relevance of previously explored PDE3A inhibitor compounds.

### Nauclefine promotes interaction between PDE3A and SLFN12

**Fig. 4:**
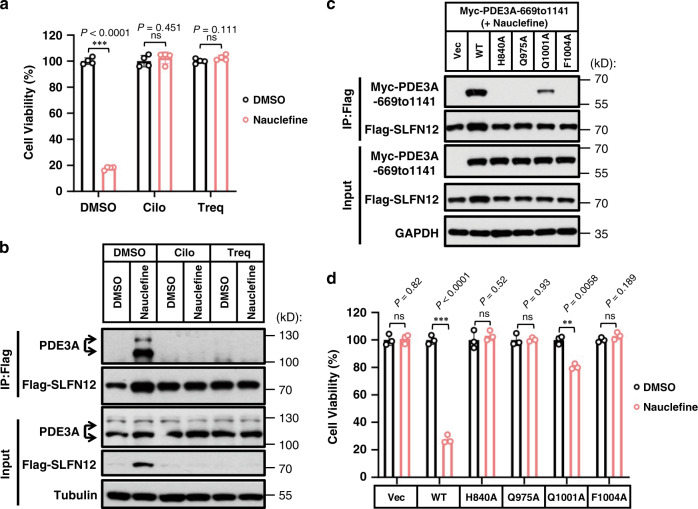
Interaction between SLFN12 and PDE3A is required for nauclefine-induced cell death. **a**, **b** The PDE3 inhibitors cilostazol (1 µM) or trequinsin (50 nM) were co-treated with nauclefine (200 nM) in HeLa cells with Flag-SLFN12 stably expressed. ATP levels were measured to assess cell viability **a** (*n* = 4, examined in three independent experiments). This is a representative result (mean ± SD) and the *p* values from Student’s two-tailed unpaired *t*-tests are indicated. *p < 0.05; **p < 0.01; ***p < 0.001; ns not significant. Cells were treated as in **a** for 20 h, followed by immunoprecipitation (**b**). Cilo denotes cilostazol, and Treq denotes trequinsin. **c** Flag-SLFN12 was transfected into HeLa cells stably expressed WT or mutant PDE3A. Nauclefine (200 nM) was then administered and cells were treated for 20 h, followed by immunoprecipitation. **d** The indicated cells were treated by nauclefine (200 nM) for 36 h, followed by assessment of cell viability based on cellular ATP levels (*n* = 3, examined in two independent experiments). The western blots are representative of results from two independent experiments.

We performed immunoprecipitation experiments with extracts from HeLa cells to show that PDE3A’s binding to nauclefine promotes its interaction with SLFN12 and we found that this nauclefine-induced PDE3A–SLFN12 interaction was blocked by co-treatment with the previously known noncytotoxic PDE3A inhibitors cilostazol and trequinsin (Fig. 4a, b); cilostazol and trequinsin blocked nauclefine–PDE3A binding (Supplementary Fig. 7c), a finding similar to reports about E2^19^ and DNMDP^13^. The nauclefine-induced PDE3A–SLFN12 interaction was also reduced by PDE3A mutations including H840A, Q975A, Q1001, and F1004A (Fig. 4c), and we found that this decrease in the extent of the interaction caused compromised cell death outcomes (Fig. 4d). Similarly, these PDE3A mutation variants lost the ability to bind SLFN12 after DNMDP treatment, and could not mediate DNMDP induced cell death (Supplementary Fig. 8a, b).

These results suggest that nauclefine initially binds with PDE3A, which then somehow induces interaction between PDE3A and SLFN12. The amino acids (H840A, Q975A, Q1001A, and F1004A) involved in both nauclefine–PDE3A and PDE3A–SLFN12 binding in a direct or indirect manner. It bears emphasis that both interactions are essential for nauclefine-induced PDE3A- and SLFN12-dependent cell death. Further supporting this, we found that the I105N SLFN12 variant lost the ability to interact with PDE3A upon nauclefine treatment (Supplementary Fig. 8c), explaining its disability in mediating nauclefine-induced cell death (Supplementary Fig. 6c).

### Increased SLFN12 stability promotes cell death

**Fig. 5:**
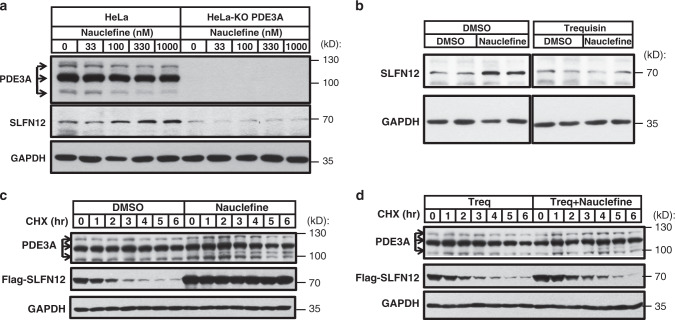
Nauclefine caused increased SLFN12 protein stability. **a** Indicated concentrations of nauclefine were used to treat HeLa or PDE3A-KO HeLa cells for 24 h, then cells were collected for western blot analysis. **b** Nauclefine (250 nM) or nauclefine (250 nM) plus trequinsin (50 nM) were used to treat HeLa cells for 24 h, then cells were collected for western blot analysis. Nauclefine (200 nM) (**c**) or nauclefine (200 nM) plus trequinsin (50 nM) (**d**) were used to treat into Flag-SLFN12 stable cells for 24 h. Treq denotes trequinsin. Cells were then treated with 100 µg/mL CHX for the indicated times and subjected to western blot analysis. The western blots are representative of results from two independent experiments.

Nauclefine treatment of WT HeLa cells increased endogenous SLFN12 protein levels (Fig. 5a), and this nauclefine-induced increase did not occur in PDE3A knockout cells (Fig. 5a). The increase was also blocked by the PDE3A inhibitor trequinsin (Fig. 5b), indicating that nauclefine increased protein level of SLFN12 in a PDE3A-dependent manner. Confirming that altered SLFN12 protein stability does mediate nauclefine’s effects, treatment of cells with cycloheximide (CHX) to block protein synthesis^35^ revealed that the typically rapid decay of the signal for SLFN12 (with half-life less than 2 h) could be substantially extended by co-treatment with nauclefine (Fig. 5c). In light of our aforementioned finding that trequinsin blocks the nauclefine-induced PDE3A–SLFN12 interaction, it was consistent when we found that co-treatment of cells with trequinsin and nauclefine resulted in the recovering rapid SLFN12 decay phenotype (Fig. 5d). Our results indicated that nauclefine binds PDE3A and induces recruitment of SLFN12, which increased SLFN12 stability by preventing its fast decay.

To assess how increased SLFN12 protein levels affect cells, we overexpressed SLFN12 by transfection with mCherry-SLFN12 or by Dox-induced expression of SLFN12 in SLFN12-KO cells. Transfection of mCherry-SLFN12 caused apoptotic morphology and cell death (Supplementary Fig. 9a, b). Overexpression of SLFN12 in SLFN12-KO HeLa cells also caused time-dependent and SLFN12 dose-dependent cell death (Supplementary Fig. 9c, d). In addition, nauclefine increased the SLFN12 protein level and decreased the protein level of the antiapoptotic protein, BCL-2, and did so in a PDE3A-dependent manner (Supplementary Fig. 9e). Overexpression of SLFN12 by a Dox-inducible system also decreased the protein level of Bcl-2 (Supplementary Fig. 9f). Our results together establish that an increased SLFN12 protein level is sufficient to mimic the nauclefine-induced increased SLFN12 level and to cause cell death.

### Nauclefine inhibits tumor growth in vivo

**Fig. 6:**
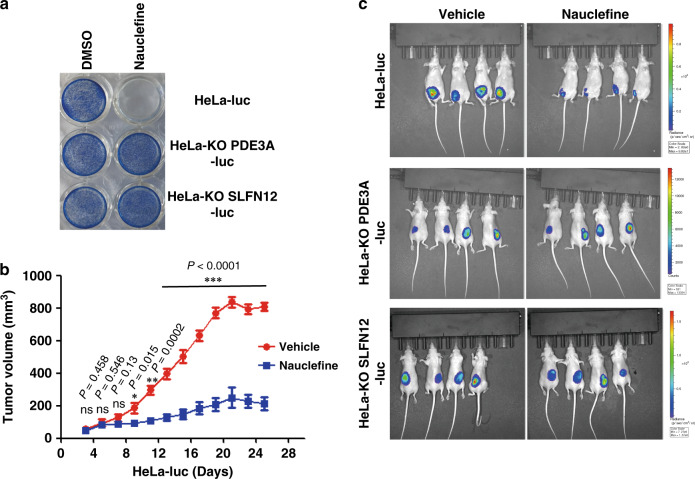
Nauclefine inhibits growth of human tumor xenografts in mice. **a** Nauclefine (200 nM) was used to treat HeLa-luc, HeLa-KO PDE3A-luc, or HeLa-KO SLFN12-luc cells for 48 h, then cells were stained by methylene blue. **b** Mice implanted with WT HeLa cells were intratumorally injected with vehicle (*n* = 4) or nauclefine (*n* = 5) for 22 days. Tumor volumes were measured on the indicated days. Student’s *t* test (two-tailed, unpaired) was performed with mean ± SD. *p < 0.05; **p < 0.01; ***p < 0.001; ns not significant. **c** Mice (*n* = 4) implanted with WT or mutant HeLa cells were intratumorally injected with vehicle or nauclefine. The luciferase substrate was intraperitoneally injected to visualize tumor size.

As nauclefine caused apoptotic cell death in in vitro cultured cells, we investigated its antitumor potential in vivo. We adopted a tumor size assessment strategy based on stably expressing luciferase in HeLa cells (Supplementary Fig. 10a), which enables assessment of relative cell numbers upon the intraperitoneal injection-based addition of the luciferase substrate to tumor-bearing mice (Fig. 6c and Supplementary Fig. 10a). After confirming that expression of the luciferase gene does not alter HeLa cell sensitivity to nauclefine (Fig. 6a), the HeLa-luc, PDE3A-KO, or SLFN12-KO cells were subcutaneously implanted into immunodeficient mice, respectively. Nauclefine is rapidly degraded in blood and liver when intraperitoneal injected or orally administrated in our preliminary attempts. Therefore, we employed the intratumoral injection to explore the antitumor potential of nauclefine. Once the implanted cells had developed into tumors after 3 days (tumor volume ~60 mm^3^), nauclefine was injected once per day for 22 days. Our results showed that nauclefine significantly reduced overall growth of tumor from HeLa-luc cells (Fig. 6b), with significant reduction in tumor volume apparent after treatment day 6 (Fig. 6b). Moreover, the inhibition of tumor growth by nauclefine was dependent on the expression of both PDE3A and SLFN12 (Fig. 6c). Notably, nauclefine treatment did not cause any reduction in the body weights of the mice (Supplementary Fig. 10b). Our results highlighted the essentiality of both PDE3A and SLFN12 in an in vivo context.

We also tested nauclefine against other PDE3A-expressing cancer cell lines, including MCF7, EKVX, and H4 cells. Nauclefine caused cell death of the MCF7, EKVX, and H4 lines, and the cell death could be blocked by the noncytotoxic PDE3 inhibitors cilostazol and trequinsin (Supplementary Fig. 11a–c). Furthermore, knockdown of PDE3A expression in MCF7, EKVX, and H4 cells also blocked nauclefine-induced cell death (Supplementary Fig. 11d–f), and blocked the nauclefine-dependent increase in the SLFN12 protein level (Supplementary Fig. 11g). And nauclefine also reduced the growth of xenograft tumors established from H4 cells, doing so in a PDE3A-dependent manner (Supplementary Fig. 11h, i). Together, these results highlight the apparently quite general anticancer potential of nauclefine against PDE3A-expressing cells.

## Discussion

In conclusion, we revealed the potential of an indole alkaloid natural product as a lead compound for anticancer therapy. We found nauclefine efficiently induces apoptosis of HeLa cells which is fully dependent on binding of nauclefine with PDE3A. Among all of the known PDE3A modulators that can induce PDE3A- and SLFN12-dependent apoptosis, nauclefine is a compound which does not inhibit the PDE activity of PDE3A; it therefore represents a kind of small molecule for inducing PDE3A- and SLFN12-dependent apoptosis that does not affect the canonical function of PDE3A.

Note that our conclusion regarding the PDE3A H840A mutant is at least partially inconsistent with a recently published result^22^: in their experiments, they found that, as compared with WT PDE3A protein, a 3 μl of DNMDP-2L resin bound a reduced amount of V5-tagged PDE3A H752A, H840A, D950A, and F1004A truncation variants (aa 668–1141). However, their experiments showed a similar decrease between the H840A and F1004A variant proteins, unlike our results showing a larger extent of decrease for the F1004A variant protein over the H840A variant. We speculate that a reason for this apparent different may originate from their use of 200 μg of input proteins compared with the 5000 μg proteins that we used as input. An excess of prey proteins could cause more proteins (PDE3A H840A variant) binding to DNMDP-2L resin present, even when the binding ability of PDE3A H840A variant protein was only slightly stronger compared with PDE3A F1004A variant protein.

In this study, we used the method “intratumoral injections” to investigate the essentiality of both PDE3A and SLFN12 in an in vivo context considering the fact that nauclefine is rapidly degraded when intraperitoneal injected or orally administrated in mice. Further improving the efficiency and stability this molecule would be urgent to push nauclefine towards clinical evaluation. Although nauclefine is at present a medical tool, we are primarily excited about the scientific implications of the discoveries as summarize below.

Regarding biochemical mechanisms: previous studies have reported that some PDE3 enzymatic inhibitors of (like DNMDP and anagrelide) activate PDE3A- and SLFN12-dependent cell death, but some PDE3 inhibitors (like cilostazol and trequinsin) do not activate, but rather block cell death as induced by PDE3A inhibitors. Reasonably based on their data, these studies concluded that the death is not caused by simple inhibition of its PDE3A enzyme activity. However, whether or not cell death still occurs when the PDE activity of PDE3A is preserved has never been explored or understood. We show that a PDE3A modulator that binds PDE3A can initiate PDE3A- and SLFN12-dependent cell death without affecting the PDE activity of PDE3A, thus establishing that pathway activation does not require the inhibition of the PDE activity of PDE3A.

Regarding clinical applications: there is now strong clinical evidence supporting that targeting PDE3 is viable for treatment of intermittent claudication, thrombocythaemia, and heart failure. Other studies, as well as our present study, indicate the potential of the PDE3-targeting strategy for developing anticancer therapies. Notably, inhibition PDE3A’s PDE activity results in side effects like headache, diarrhea, unusual weakness/fatigue, hair loss, nausea, dizziness, and cardiac dysrhythmias, as well as affecting oocytes maturation, decreasing platelet formation, and increasing the risk of sudden death in patients with cardiac diseases; these side effects have greatly limited drug development efforts for targeting PDE3^36^. Our discovery and in vivo efficacy demonstration of an agent that can inhibit tumor growth in a PDE3A-dependent manner without affecting the enzyme activity of PDE3A supports the clinically attractive idea that using a PDE3-targeting strategy to develop anticancer therapies may preclude some of the side effects known to accompany chemical inhibition of PDE3A’s PDE activity.

Thus, a highly promising purport of our work with this scientific tool molecule is support for a line of thinking about PDE targeting, inviting scientists to shift their thinking away from only enzyme inhibition and expanding the scope to include potential enzyme-independent mechanisms that can be studies and potentially exploited to develop novel therapies. To our understanding at least, this is a highly innovative line of thinking, and it is based on the PDE modulator that exerts its effects without affecting enzyme activity. It also bears emphasis that our work supports being cautious before initiating any clinical trials to target PDE for intervening in disease: in light of our discoveries, future research should take care to establish whether or not a given inhibitor-PDE interaction is enzyme activity dependent. If it is not, then developing molecules may avoid some of the known side effects caused by inhibition PDE phosphodiesterase activity. We anticipate that knowing this information about a given compound might improve the success rates of PDE related clinical trials; many PDE-targeting clinical trials attempted to date have failed because of additional safety concerns arisen even those molecules passed phase I clinical trials^36^.

## Methods

### Cells and plasmids

HeLa, H4, 293T, MCF7, EKVX, SGC-7901, and A549 cells were cultured in DMEM (GIBCO) with 10% FBS (Invitrogen) and 1% penicillin and streptomycin at 37 °C with 5% CO_2_. psPAX2, pMD2.G, pWPI, pFatBac-HTA, and pX458 constructs were kept in our lab. Myc-tagged full-length PDE3A and HA-3xFlag tagged SLFN12 were constructed in pWPI. The pLKO.1-shPDE3A plasmids were from Merck (TRCN0000048784). Point mutations were generated using a Quikchange Site-Directed Mutagenesis Kit. Truncated PDE3A (aa 669 to 1141) and the corresponding point mutations were also contructed in the pWPI vector with a Myc tag fused to the C-terminal of PDE3A. mCherry or mCherry-SLFN12 were inserted in the pWPI vector. Truncated PDE3A (aa 669–1141) cDNAs were inserted into the pFastBac HTA with a 6xHis tag fused to the N-terminus.

### Cell viability assay

Cell viability assays were performed by measuring the cellular ATP level using a Cell Titer-Glo Luminescent Cell Viability Assay kit (Promega) according to the manufacturer’s instructions. The luminescence intensity was read by a microplate reader (Tecan GENios).

### Western blotting

Cells were lysed either in Flag-lysis buffer or directly solubilized in 1× SDS buffer, then subjected to SDS-PAGE. Cells lysed with Flag-lysis buffer were set on ice for 30 min, and then centrifuged at 20,000 × *g* for 10 min. The supernatants were then mixed with 4× SDS buffer and subjected to SDS-PAGE. The antibodies used in this research were: PDE3A antibody from Bethyl Laboratories (1:1000, Cat# A302-740A); SLFN12 antibody from abcam (1:400, Cat# ab234418); Cleaved-Caspase-3 antibody from Cell Signaling technology (1:1000, Cat# 9661); Caspase-9 antibody from Cell Signaling technology (1:1000, Cat# 9502); PARP antibody from Cell Signaling technology (1:1000, Cat# 9542); Bcl-2 antibody from Cell Signaling technology (1:1000, Cat# 4223); anti-Rabbit-HRP antibody from Sigma-Aldrich (1:5000, Cat# A0545); anti Mouse-HRP antibody from Sigma-Aldrich (1:5000, Cat# A9044); MYC-HRP antibody from MBL (1:1000, M-047-7); Flag-HRP antibody from Sigma-Aldrich (1:10,000, Cat# A8592); Actin-HRP antibody from MBL (1:50,000, Cat# PM053-7); α-Tubulin-HRP antibody from MBL (1:50,000, PM054-7); and anti-GAPDH-HRP antibody from MBL (1:50,000, Cat# M171-1).

### Compound screen assay

Compound screens were performed at the chemistry center at the National Institution of Biological Science, Beijing. HeLa cells (2000 cells per well) were seeded in a 384 well plate on day 1. On day 2, about 3000 compounds from the FDA-approved drug sub-library were individually added in each well to a final concentration of 6 µM. Then, 250 nM of nauclefine was added into each well. Cell viability was assessed 48 h after treatment. Hits with significant protection were selected for further verification.

### Purification of proteins

The cDNAs of WT or site-specific mutants of PDE3A (amino acid 669–1141) were inserted into pFastBac HTA with a 6xHis tag fused at the N-termini of the proteins. pFastBac HTA-PDE3A vectors were transformed into DH10Bac cells (Weidi biology) to generate a recombinant baculovirus genome which was later transfected into Sf21 cells. Insect virus infected Sf21 cells were pelleted and broken with French press in buffer containing 20 mM Tris-HCl, pH 8.0, 150 mM NaCl, and 15 mM imidazol. Cell debris was discarded by centrifugation and supernatants were passed through a nickel column, a Q column, and a superdex 200 column, and were finally stored in buffer containing 20 mM Tris-HCl, pH 8.0, and 150 mM NaCl. The purity of purified proteins was assessed with coomassie blue staining.

### DNMDP-2L pull down

DNMDP-2L resin was synthesized in the Chemistry Center in National Institute of Biological Sciences, Beijing. Purified PDE3A proteins or cell lysates were mixed with competitors for 30 min. DNMDP-2L resin was then added into the mixture overnight. Unbound proteins were washed five times with PBS or Flag-lysis buffer. Bound PDE3A proteins were analyzed by immunoblotting.

### HeLa xenograft models

HeLa-luc, HeLa-KO PDE3A-luc, or HeLa-KO SLFN12-luc cells (5 × 10^6^ per mouse) mixed with Matrigel (Corning) were subcutaneously injected into female nude mice (Balb/c-nude, 6–7 week). Mice were maintained in an animal facility with 12 h light/12 h dark cycles, temperature (22–24 °C), humidity (40–60%) at the National Institute of Biological Sciences, Beijing. When the tumor volume reached about 60 mm^3^, vehicle (10% DMSO, 30% PEG-4000, 60% Saline) or nauclefine (5 mg/kg) were intratumorally injected once per day for 22 days. Tumor volumes were calculated as (length × width^2^)/2. d-Luciferin (15 mg/mL in DPBS, 200 μL for each mouse) was injected to visualize tumor size in vivo. Photographs were captured under IVIS Spectrum. Animal experiments were conducted following the Chinese Ministry of Health national guidelines and performed in accordance with institutional regulations reviewed and approved by the Institutional Animal Care and Use Committee of the National Institute of Biological Sciences.

### Confocal microscopy

Cells transfected with PLCD1-GFP for 12 h were reseeded in 35 nM glass-bottom culture dishes (MatTek), then treated with the agents indicated in the relevant figure captions. For mCherry/mCherry-SLFN12 expression, vectors were transfected in cells seeded in 35 nM glass-bottom culture dishes for 36 h. Images were captured using a Nikon A1 microscope.

### Flow cytometry

Lentivirus-infected cells were sorted based on expression of GFP in successfully infected cells. Apoptotic cells were counted after staining using an Annexin V-FITC/PI Apoptosis Assay Kit (DOJINDO) according to the manufacturer’s instructions. Cells were passed through a 40 µM griddle to discard clustered cells before being analyzed on a BD FACSAria III flow cytometer. Data were processed using the software FlowJo.

### Transfection and virus packaging

Plasmids were transfected into cells with Lipofectamine 3000 according to the manufacturer’s instructions. Genes inserted into lentiviral based vectors were co-expressed with pMD.2G and psPAX2 in 293T cells for 48 h. Supernatant (containing virus particles) was filtered through a 0.22 µM filter and used to infect cells with polybrene (5 µg/mL). Positively infected cells expressed GFP and were sorted by FACS to establish cell lines stably expressing specific genes.

### Immunoprecipitation

Cells were lysed in Flag-lysis buffer containing 50 mM Tris-HCl, pH 8.0, 137 mM NaCl, 1 mM EDTA, 1% Triton-X100, 10% glycerol, and protease inhibitor cocktail (Roche) on ice for 30 min. Cell debris was discarded after centrifugation at 20,000 × *g* for 10 min. The soluble fraction was incubated with Flag-M2 beads (Sigma) overnight. Unbound proteins were washed five times with Flag-lysis buffer. Immunoprecipitated proteins were either eluted with the 3xFlag peptide (1 mg/mL) or directly boiled in 1× SDS buffer. Bound proteins were analyzed by western blotting.

### CHX stability assay

Cells were treated with CHX for the times listed in the relevant figure captions. Cell pellets were then collected and lysed in 1× SDS buffer. The protein level in cell samples was analyzed by western blotting.

### Methylene blue staining

Cells were seeded in six-well plates. After treatment, cells were washed with PBS and stained with 1.5% methylene blue dissolved in 50% ethanol for 10 min at room temperature. Cells were then washed with PBS and photos were taken.

### Phosphodiesterase assay and LC–MS/MS analysis

A total of 10 ng purified PDE3A protein was incubated with 10 µM cAMP (Sigma) in the presence of increasing concentrations of trequinsin or nauclefine (indicated in Fig. 3) in the buffer used for the PDE assays at room temperature for 90 min. A tenfold excess of methanol was added to the reaction mixture, followed by centrifugation at 20,000 × *g* for 10 min. The supernatant was then analyzed via LC–MS/MS.

LC–MS/MS analysis was performed using an Agilent 1290 Infinity UHPLC coupled to an Agilent 6495 Triple Quadrupole mass spectrometer operated in multiple reaction monitoring (MRM) mode. The separation was carried out by a Waters ACQUITY UPLC BEH Amide column (2.1 mm × 100 mm, 1.7 µm), heated at 35 °C. The mobile phase consisted of water with 25 mM ammonium acetate and 25 mM ammonium hydroxide (A) and acetonitrile (B) with the following gradient: 0–1 min, 85% B; 1–12 min, 85–65% B; 12–12.1 min, 65–40% B; 12.1–15 min, 40% B; 15–15.1 min, 40–85% B; 15.1–20 min, 85% B. The flow rate was set at 0.3 mL/min. Overall, 1 µl sample was injected. The MRM transitions were m/z 330.1 → m/z 118.6 (CE 50V) and m/z 330.1 → m/z 135.8 (CE 33V) in positive ionization mode. The concentration was calculated from external calibration curves constructed using a serial dilution of adenosine 3′,5′-cyclic monophosphate solutions, ranging from 1 to 500 ng/mL. Data processing was performed with Agilent MassHunter software (Ver. B.07.00).

To nauclefine, The separation was carried out by a Agilent Zorbax Eclipse Plus C18 column (2.1 mm × 50 mm, 1.8 µm), heated at 35 °C. Mobile phase consisted of water with 0.1% formic acid (A) and acetonitrile with 0.1% formic acid (B) with the following gradient: 0–1 min, 5% B; 1–4 min, 5–95% B; 4–5 min, 95% B; 5–5.01 min, 95–5% B; 5.01–7 min, 5% B. The flow rate was set at 0.3 mL/min. Overall, 2 µl sample was injected. The MRM transitions were m/z 288.12 → 273.1(CE 39 V) and m/z 288.12 → 286.2(CE 43V) in positive ionization mode. The concentration was calculated and data were processed as described above.

### Reporting summary

Further information on research design is available in the Nature Research Reporting Summary linked to this article.

## Supplementary information

Supplementary Information

Reporting Summary

## Data Availability

There are no restrictions on data availability. All of the relevant data are available upon requesting from the lead corresponding author, Y.A. All raw blots corresponding to the SDS-PAGE gels (Figs. 1d, 2e–f, 3a–b, 4b–c, 5a–d, Supplementary Figs. 6b, 6d, 7c, 8a, 8c, 9c–f, 11g) and remaining primary data of interest (Figs. 1a, 2c, d, 3b–d, 4a, d, 6b, Supplementary Figs. 3–6a, c, 7c, 8b, 9b–d, 10b, 11a–f, h) are included in the Source Data file.

## Source data

Source Data
