# Supplementary material for: An alkaloid initiates phosphodiesterase 3A–schlafen 12 dependent apoptosis without affecting the phosphodiesterase activity
Source: Nat Commun. 2020 Jun 26;11:3236. doi: 10.1038/s41467-020-17052-4 (PMC7319972; doi:10.1038/s41467-020-17052-4)
Supplement: Supplementary file 1 — Supplementary Information [file 41467_2020_17052_MOESM1_ESM.pdf]

Supplementary Information

An Alkaloid Initiates Phosphodiesterase 3A–Schlafen 12 Dependent Apoptosis without Affecting the Phosphodiesterase Activity

Ai *et al.*

Supplementary Figure 1-12

Supplementary Table

Supplementary Note

Supplementary Reference

Supplementary Figures

A

Compound	Structure	IC50 (μM)			
		HeLa	SGC-7901	MCF-7	A549
Nauclefine (1)	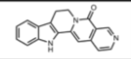	0.0052	1.07	0.186	>10
Rosettacin (2)	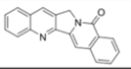	6.66	>10	>10	9.61
Naucleficine (3)	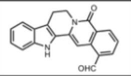	8.36	7.02	7.75	6.53
Camptothecin analog (4)	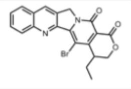	0.97	1.79	2.24	1.27
Oxypalmatine analog (5)	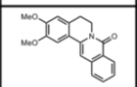	>10	>10	>10	>10
6	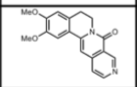	3.79	ND	ND	ND

B

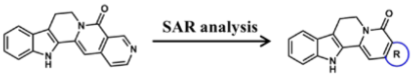

Nauclefine (1)

Compound	Structure	HeLa IC_{50} (μM)	Compound	Structure	HeLa IC_{50} (μM)	Compound	Structure	HeLa IC_{50} (μM)
1	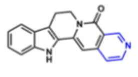	0.006	10	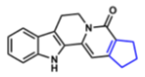	>10	14	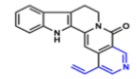	>10
7	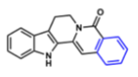	7.26	11	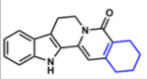	4.03	15	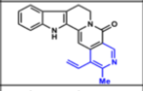	1.12
8	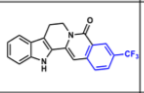	>10	12	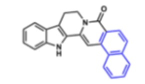	>10	16	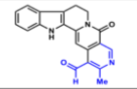	>10
9	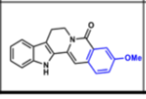	0.88	13	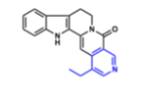	1.06	17	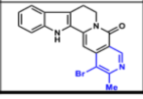	>10

Supplementary Figure 1. Nauclefine-induced cell death in HeLa cells.

(a) The compound names and structure of the indoloquinolizidine type molecules were shown. The cytotoxicity of these compounds was tested with 4 different kinds of cancer cells. (b) Structure-activity relationship studies of nauclefine were performed by varying the substitution on the core structure, and the pharmacophere optimization, and the cytotoxicity of these molecules were tested against HeLa cells.

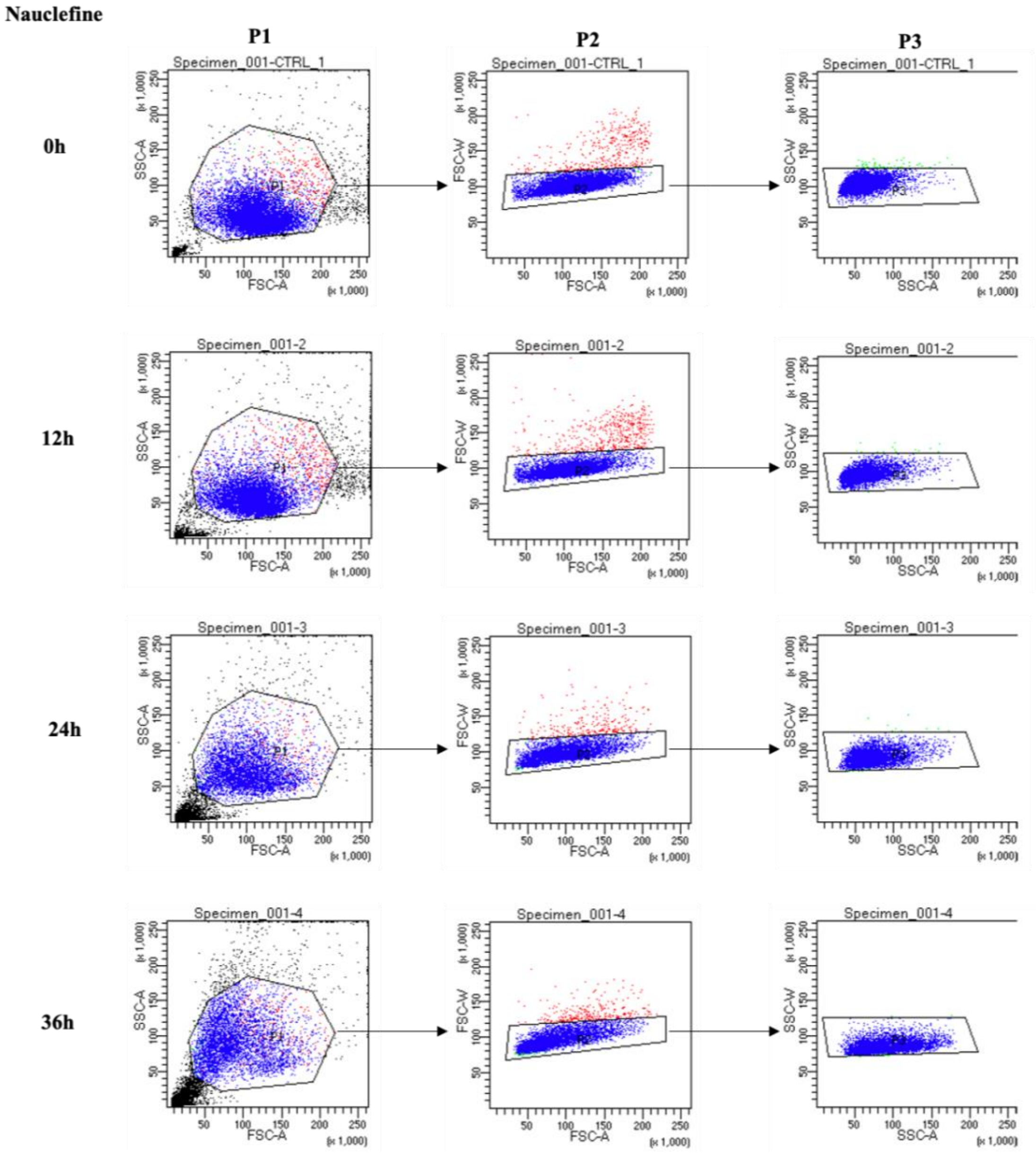

Supplementary Figure 2. Florescent activated cell sorting of nauclefine-treated HeLa cells. Nauclefine (200 nM) was used to treat HeLa cells for the indicated times. The gating strategies of flow cytometry for each time points corresponding to Fig.1C are displayed. Population 3 (P3) was selected for PI and Annexin V analysis.

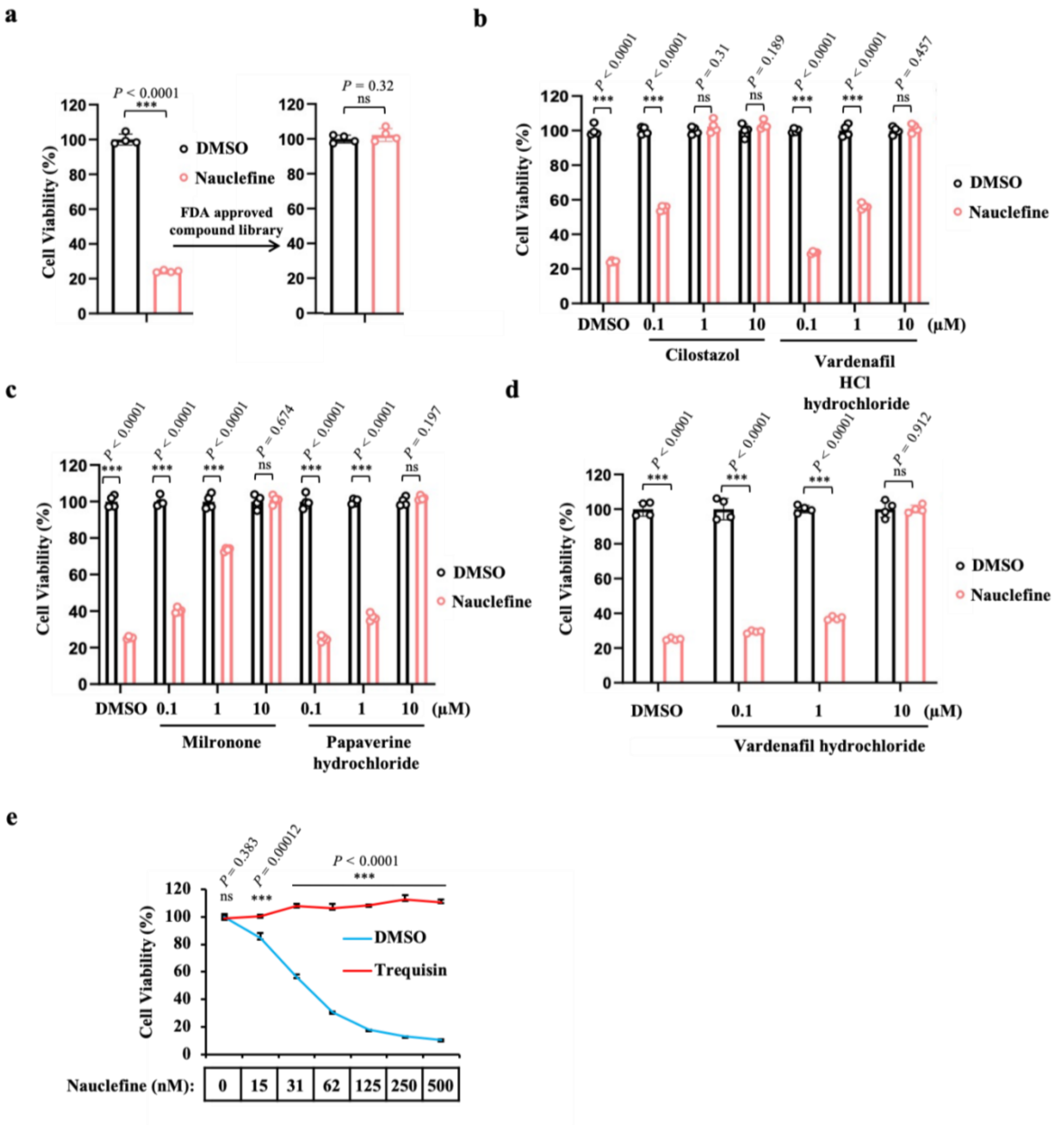

Supplementary Figure 3. PDE inhibitors block nauclefine-induced cell death.

(a) Illustration of the mechanistically annotated screen assay using the FDA-approved compound library to block nauclefine-induced cell death.

(b–d) HeLa cells were co-treated with the indicated concentrations of (b) cilostazol and vardenafil HCl hydrochloride, (c) milrinone and papaverine hydrochloride, (d) vardenafil hydrochloride and 250 nM of nauclefine for 36 h (n=4); ATP levels were then measured to assess cell viability. (e) The PDE3 inhibitor trequinsin (50 nM) was co-treated with the indicated concentrations of nauclefine in HeLa cells for 36 hours (n=4). ATP levels were measured to assess cell viability. Representative data are shown from two independent experiments as mean ± SD, and the p values from Student's two-tailed unpaired *t*-tests are indicated. *p < 0.05, **p < 0.01, ***p < 0.001, ns, not significant.

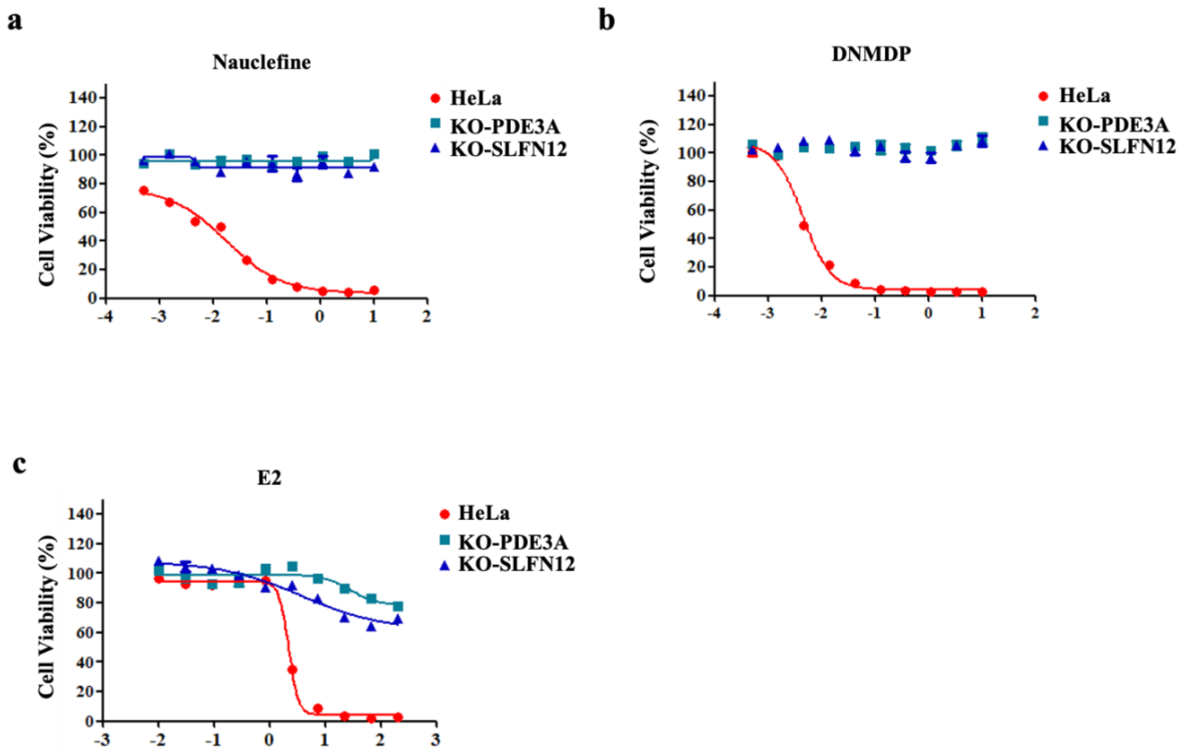

Supplementary Figure 4. Different compounds induced PDE3A-SLFN12 dependent cell death in HeLa cells.

(a–c) WT, PDE3A-KO, and SLFN12-KO HeLa cells were treated with the indicated concentrations of nauclefine (a), DNMDP (b), or E2 (c) for 48 hours. Cell viability was determined by measuring ATP levels. Representative data are shown from n=2 wells, examined over three independent experiments.

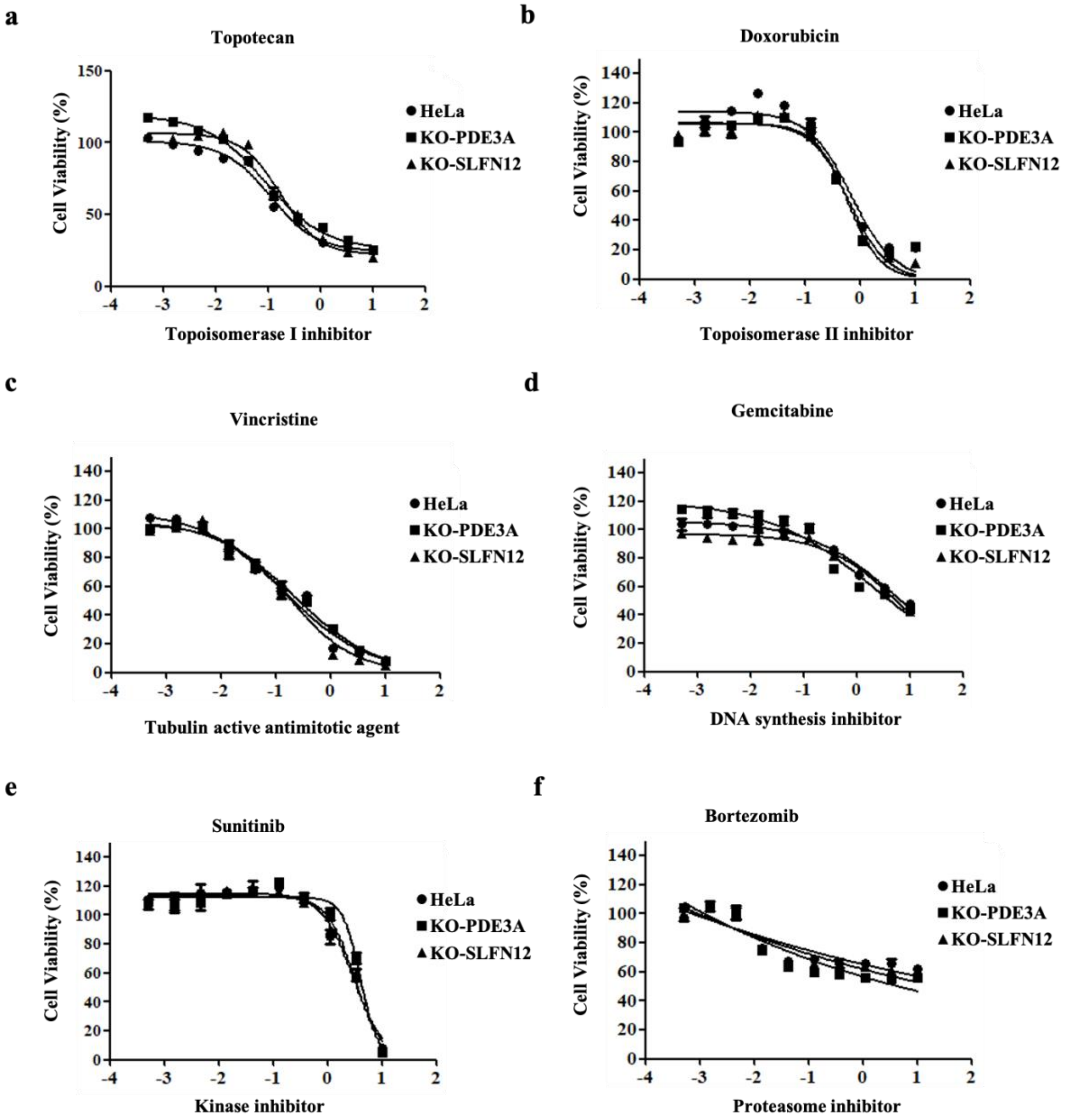

Supplementary Figure 5. Clinically used anti-cancer agents did not induce PDE3A-SLFN12 dependent cell death.

(a–f) WT, PDE3A-KO, and SLFN12-KO HeLa cells were treated with the indicated concentrations of the topoisomerase I inhibitor topotecan (a), the topoisomerase II inhibitor doxorubicin (b), the tubulin-active antimitotic agent vincristine (c), the DNA synthesis inhibitor gemcitabine (d), the kinase inhibitor sunitinib (e), or the proteasome inhibitor bortezomib (f) for 48 hours. Cell viability was assessed by measuring ATP levels. The results are representative of n=2 wells, examined over two independent experiments.

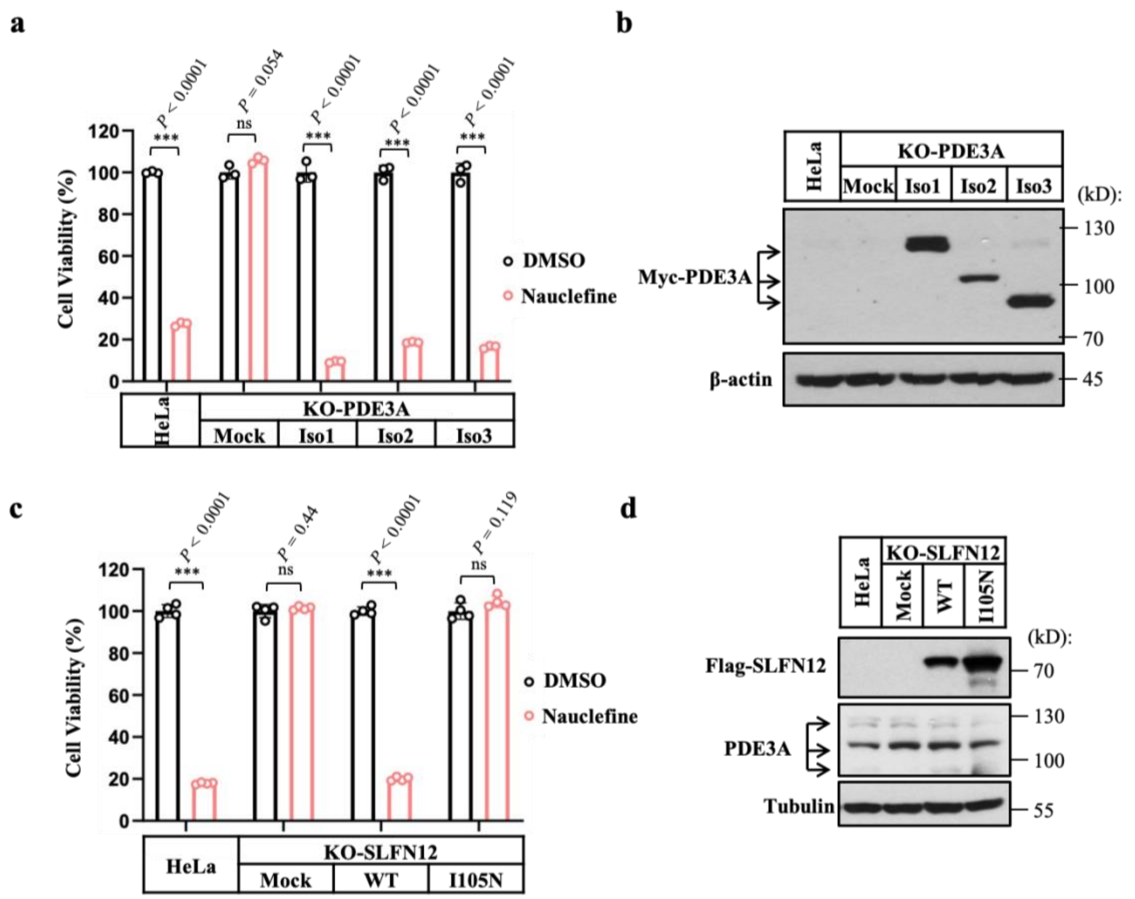

Supplementary Figure 6. PDE3A and SLFN12 are required for nauclefine-induced cell death. (a and b) C-terminal Myc-tagged Isoform 1, 2, and 3 (Iso1, 2, or 3) of PDE3A were individually expressed in PDE3A-KO HeLa cells. Nauclefine (200 nM) was administered for 36 hours (n=3). ATP levels were measured to indicate cell viability (a). Expression of PDE3A proteins were analyzed by anti-Myc antibody. β-actin was used as a loading control (b). (c and d) N-terminal Flag-tagged SLFN12 and the I105N mutant were individually expressed in SLFN12-KO HeLa cells. Nauclefine (200 nM) was administered for 36 hours (n=4). ATP levels were measured to assess cell viability (c). Expression of SLFN12 proteins were analyzed by anti-Flag antibody. Tubulin was used as a loading control (d). The western blotting was performed twice. Data are representative of two independent experiments (mean ± SD), and the p values from two-tailed unpaired Student's *t*-tests are indicated. *p < 0.05, **p < 0.01, ***p < 0.001, ns, not significant.

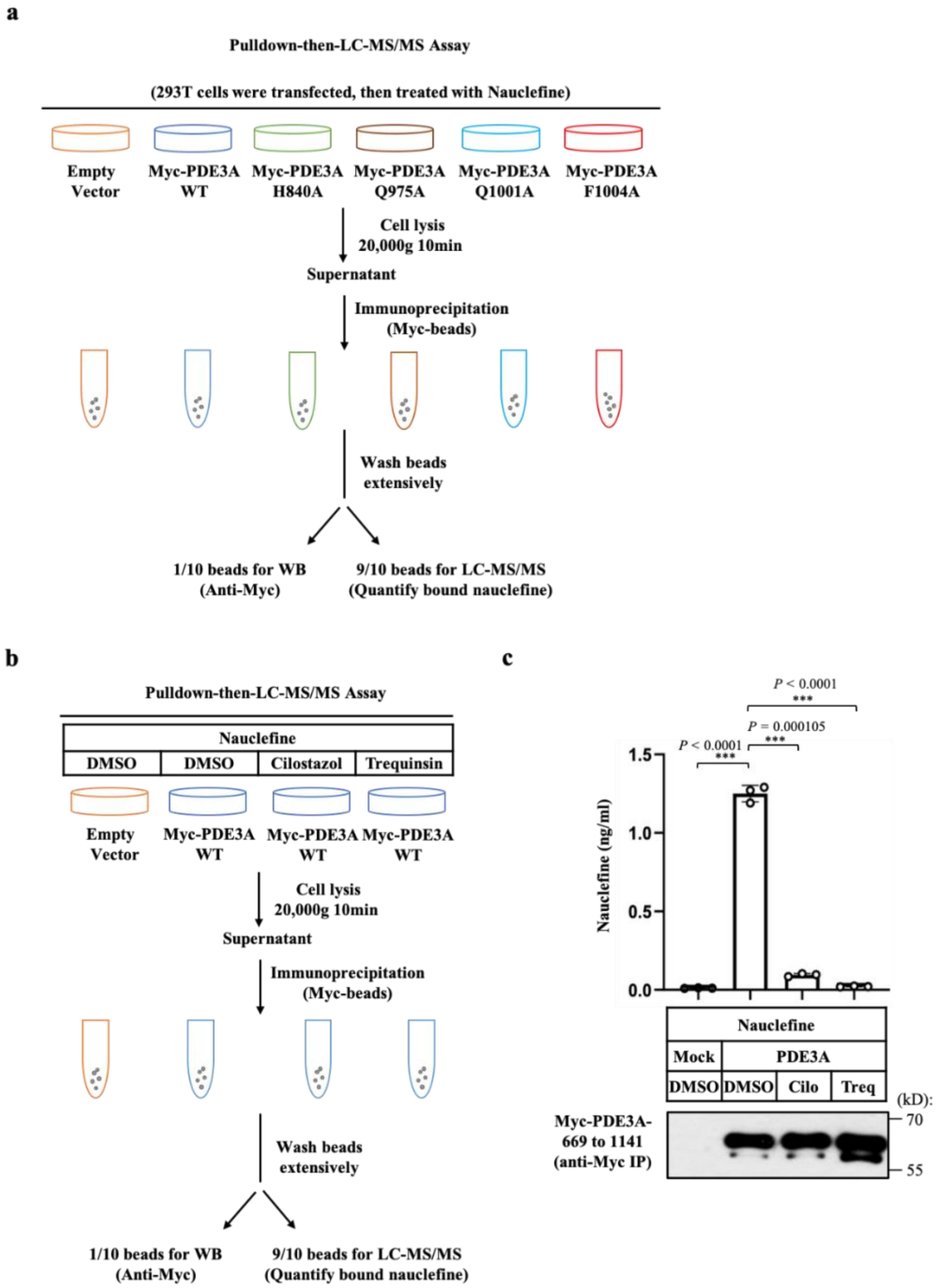

Supplementary Figure 7. Nauclefine binds PDE3A.

(a and b) The illustration of pulldown-then-LC-MS/MS assay analysis of nauclefine binding to different PDE3A mutant variants (a) and assessment of binding competition effects with the PDE3 inhibitors cilostazol and trequinsin for binding to PDE3A (b). (c) Western blotting of immunoprecipitated myc-PDE3A proteins as described in (b). The blots are representative of two independent experiments. The PDE3A-bound nauclefine was quantified by using LC-MS/MS. Cilo denotes cilostazol, and Treq denotes trequinsin. Data are from two independent experiments with n=3 replications (mean ± SD), and the p values from two-tailed unpaired

Student's *t*-tests are indicated. ***p < 0.001.

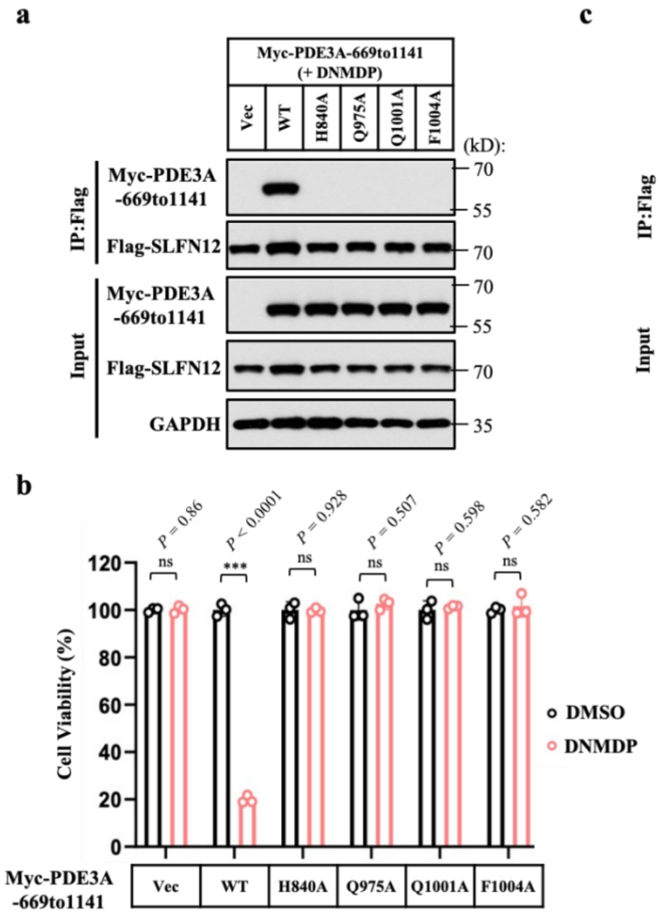

Supplementary Figure 8. Amino acids crucial for PDE3A-SLFN12 interaction.

(a) Flag-SLFN12 and WT or mutant PDE3A variants were expressed in PDE3A-KO HeLa cells. Cells were treated with DNMDP (100 nM) for 20 hours and subjected to Flag-IP. Protein levels were analyzed by western blotting. The blots are representative of two independent experiments. (b) DNMDP (100 nM) was used to treat the indicated cells for 36 hours. Cell viability was assessed by measuring ATP levels. The results are representative of three independent experiments. (c) Flag-SLFN12 WT or its I105N variant were expressed in HeLa cells for 12 hours. Then, nauclefine (200 nM) was added for 20 hours, followed by immunoprecipitation and western blotting. Representative data is shown from two independent experiments (with n=3 wells each time) as mean ± SD, and the p values from two-tailed unpaired Student's *t*-tests are indicated. *p < 0.05, **p < 0.01, ***p < 0.001, ns, not significant.

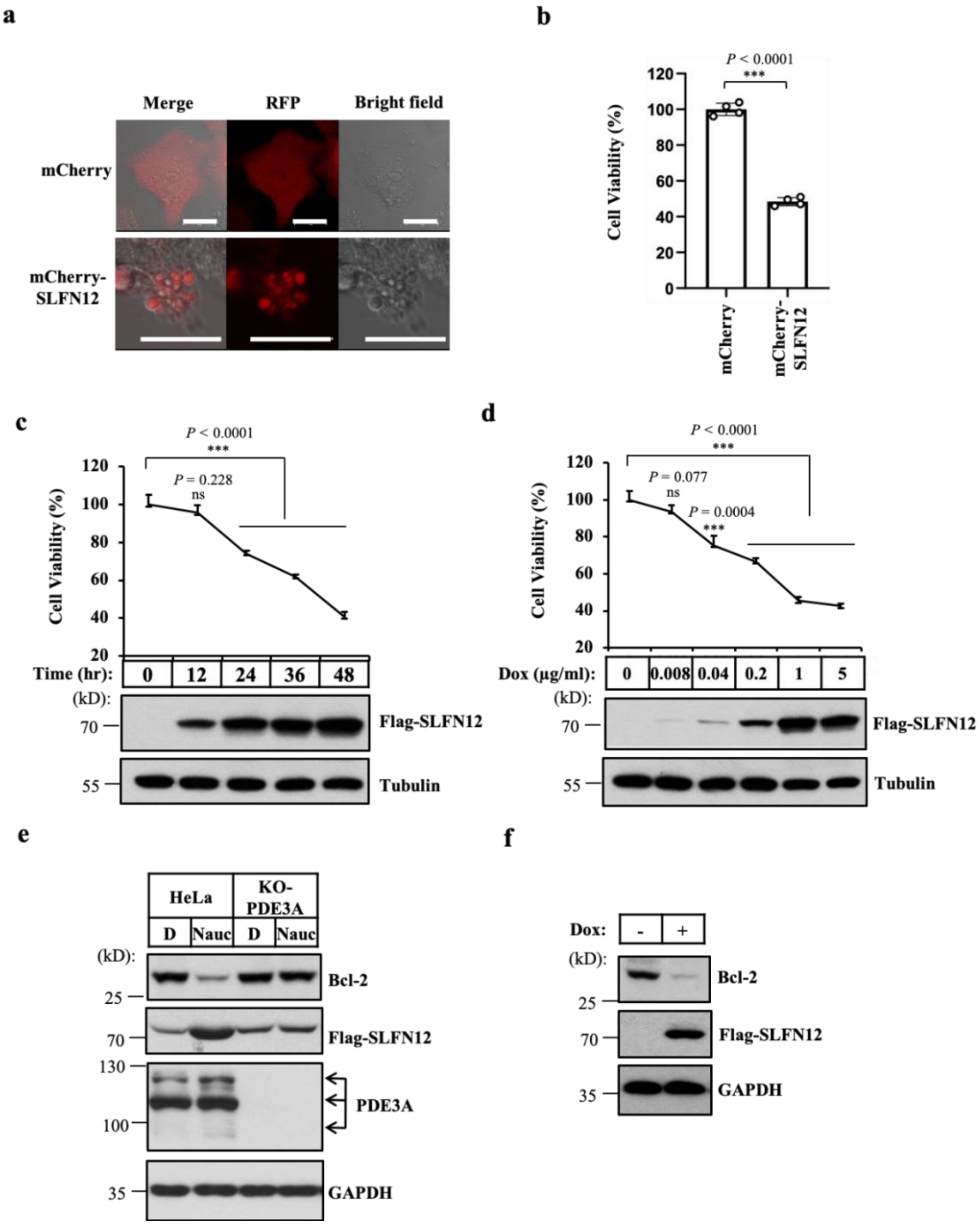

Supplementary Figure 9. Increased SLFN12 protein level induces apoptosis.

(a and b) mCherry or mCherry-SLFN12 vectors were transfected into SLFN12-KO HeLa cells for 36 hours. Cell morphology was determined using confocal microscopy (n=3 independent experiments) (a) and cell viability was assessed by measuring ATP levels (b) (n=3 independent experiments, with 4 replicates each time). Scale bars, 10 μM. (c and d) Dox (1 μg/mL) was added to drive expression of Flag-SLFN12 cells for the indicated times (c) or the indicated concentrations of Dox were added for 48 hours (d). Cell viability was determined by assessing ATP levels (n=4 wells). Expression of SLFN12 was analyzed using an anti-Flag antibody.

Tubulin was used as a loading control. Representative data are shown from two independent experiments (mean ± SD), and the p values from two-tailed unpaired Student's *t*-tests are indicated. *p < 0.05, **p < 0.01, ***p < 0.001, ns, not significant (e) Nauclefine (Nauc) decreased protein level of Bcl-2 24 hours after treatment in a PDE3A dependent manner. (f) Dox induced expression of SLFN12 decreased protein level of Bcl-2. The western blotting results were repeated twice.

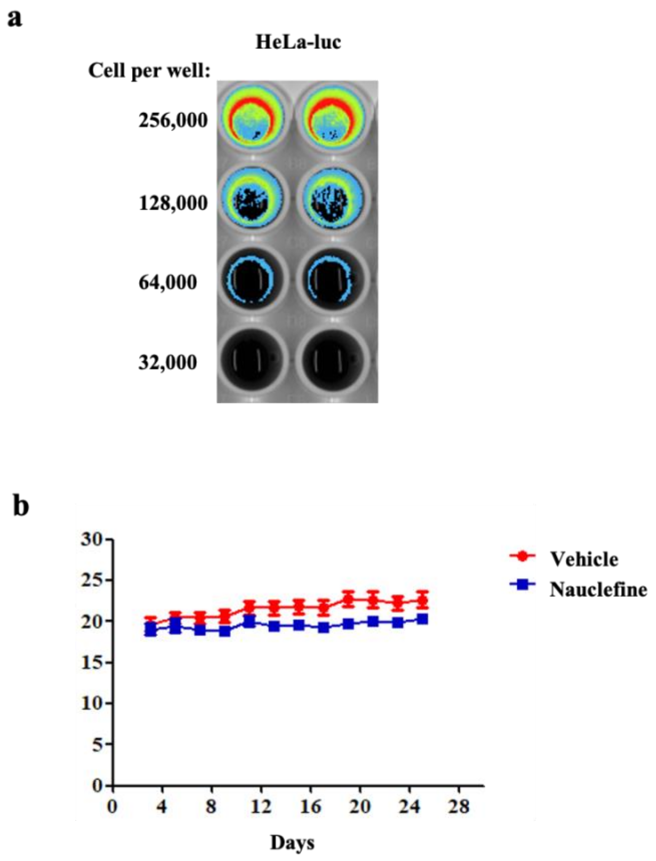

Supplementary Figure 10. HeLa-toxic nauclefine did not affect mice body weight.

(A) HeLa cells stably expressing a luciferase gene. The luciferase substrate was added to facilitate assessment of the number of HeLa-luc cells, and cell luminescence was determined.

(B) Body weights of vehicle or naulefine-treated mice (n=4) were recorded.

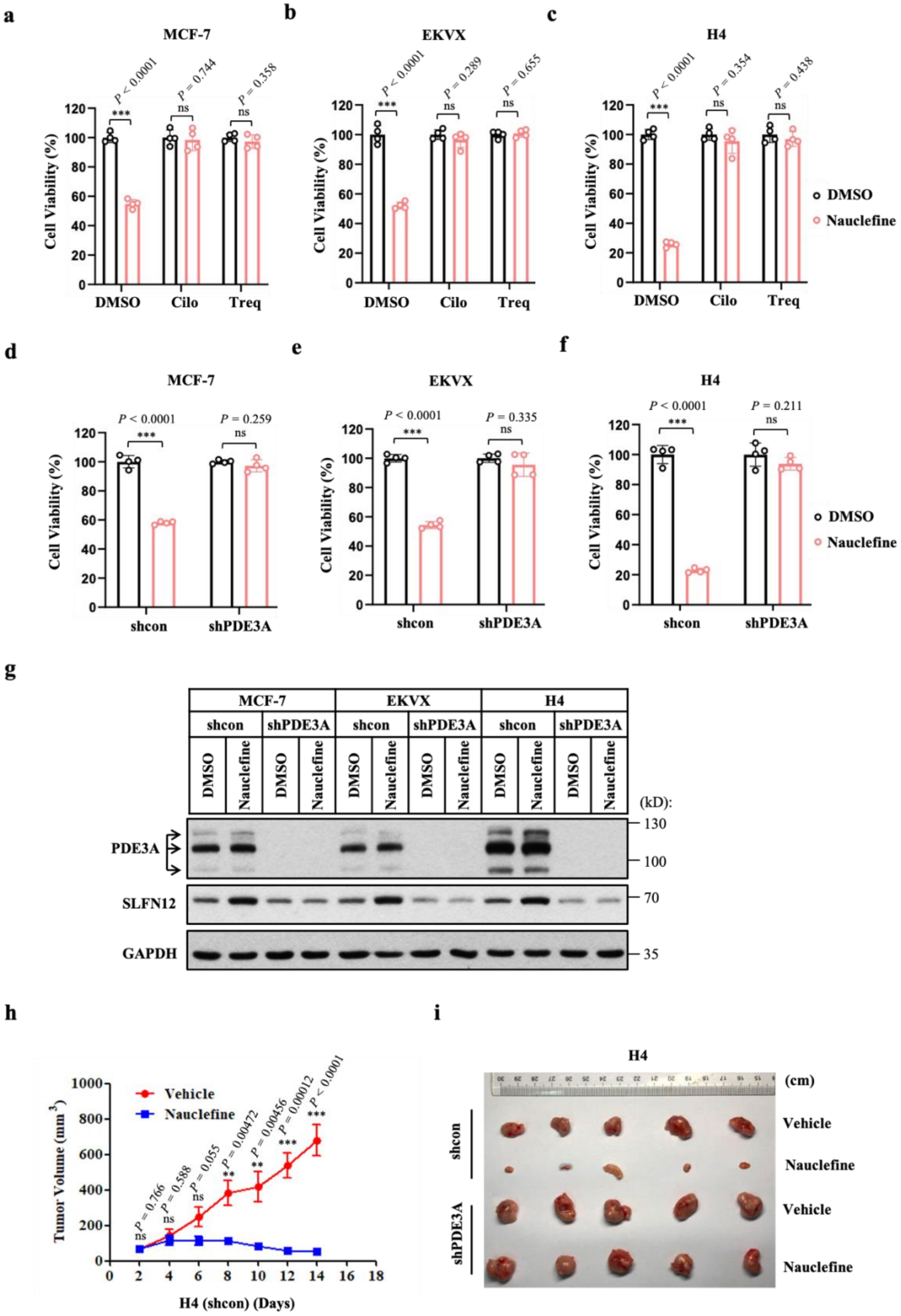

Supplementary Figure 11. Nauclefine's anti-tumor effect in other PDE3A-expressing cancer

cells.

(a~c) Cilostazol (Cilo) and trequinsin (Treq) blocked nauclefine induced cell death in MCF-7 (a), EKVX (b), and H4 (c) cells. (d~f) Knockdown expression of PDE3A in MCF-7 (d), EKVX (e), and H4 (f) cells blocked nauclefine induced cell death. Data are representative of from two independent experiments (mean ± SD), with 4 wells each time; p values from two-tailed unpaired Student's *t*-tests are indicated. *$p < 0.05$, **$p < 0.01$, ***$p < 0.001$, ns, not significant. (g) Expression of PDE3A was knocked down in MCF-7, EKVX, and H4 cells. Nauclefine (500 nM) was used to treat the MCF-7 and EKVX cells for 36 hours, and to treat the H4 cells for 24 hours. Protein expression was analyzed by western blotting with antibodies against the PDE3A and SLFN12 proteins. GAPDH was used as an internal control. Experiments were repeated twice. (h and i) Intratumoral injection of nauclefine blocked the growth of tumors (established from control shRNA H4 cells) (h), but nauclefine injection could not block the growth of tumors established from shPDE3A H4 cells (i) (n=5).

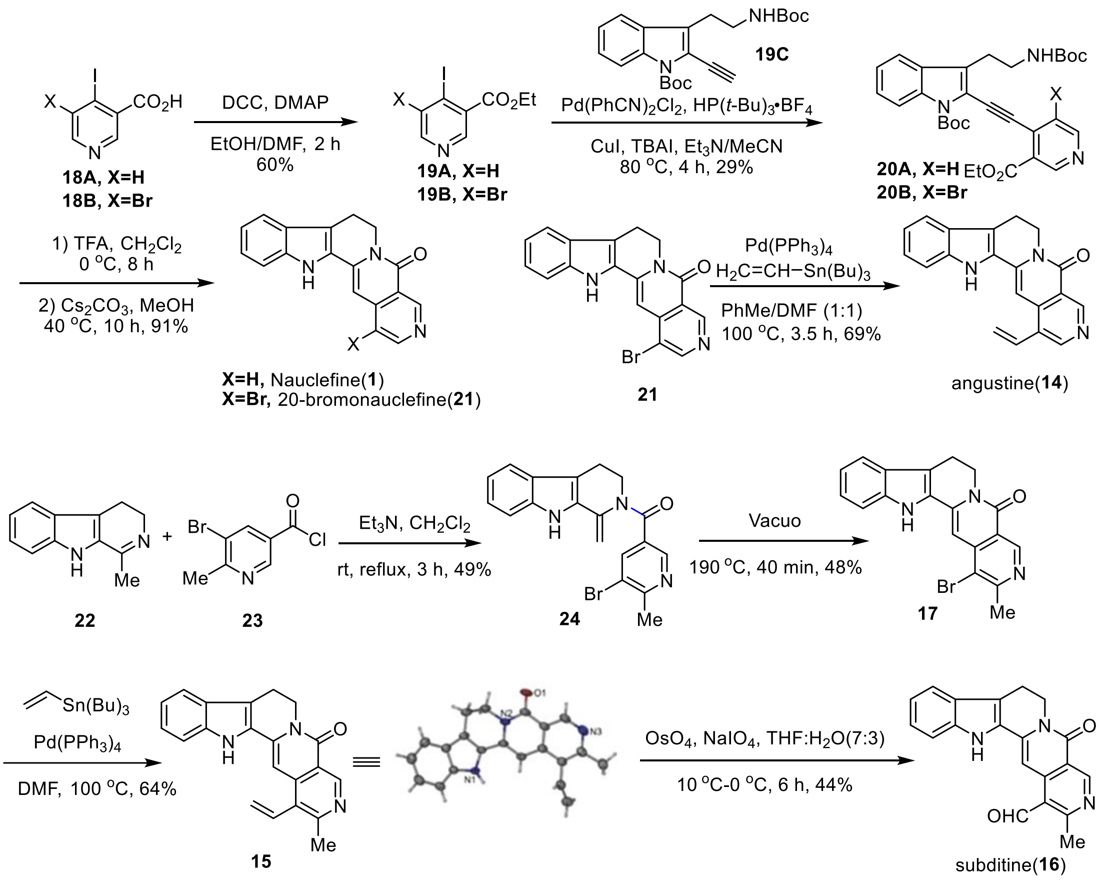

Supplementary Figure 12. Total synthesis of nauclefine, angustine, and subditine.

The indolizinone natural products nauclefine, angustine, and 20-bromonauclefine were constructed using a common cascade cyclization strategy. This process involves a key hydroamination of an internal alkyne followed by lactamization. The internal alkyne was prepared from the Sonogashira coupling of iodobenzen 19A/B and 2-ethynyl-3-aminoethyl-indole 19C. The resulting 20-bromonauclefine product from cyclization was transferred to angustine via Stille cross-coupling of a vinyl tin substrate. The subditine (16) was synthesized from oxidative cleavage of the vinyl substrate of 15, which was generated from the Stille cross-coupling of vinyl tin and bromo-methylpyridine 17. This key cyclization intermediate 17 was constructed from intramolecular cyclization of enamide 24, which was the condensation product of 5-bromo-6-methylnicotinoyl chloride 23 and imine 22.

Supplementary table

List of primers.

Name	Sequence
BamHI-PDE3A iso1-F	CGCGGATCCATGGCAGTGCCCGGCGAC
BamHI-PDE3A iso2-F	CGCGGATCCATGGTGACGATATTTTCC
BamHI-PDE3A iso3-F	CGCGGATCCATGATGACCCTCACCAAAAGC
BamHI-ATG-PDE3A aa669-F	CGCGGATCCATGAAACCAATTCTTGCTCCC
MluI-PDE3A-noTGA-R	GATAACGCGTTAACTGGTCTGGCTTTTG
PDE3A-H840A-F	GCCATGCACGATTATGATGCTCCAGGAAGGACTAATGCTT
PDE3A-H840A-R	AAGCATTAGTCCTTCCTGGAGCATCATAATCGTGCATGGC
PDE3A-Q975A-F	TGTCAATGAATTTTATGAAGCGGGTGATGAAGAGGCCAGC
PDE3A-Q975A-R	GCTGGCCTCTTCATCACCCGCTTCATAAAATTCATTGACA
PDE3A-Q1001A-F	TCCTCAGCTGGCCAACCTTGCGGAATCCTTCATCTCTCAC
PDE3A-Q1001A-R	GTGAGAGATGAAGGATTCCGCAAGGTTGGCCAGCTGAGGA
PDE3A-F1004A-F	GGCCAACCTTCAGGAATCCGCCATCTCTCACATTGTGGGG
PDE3A-F1004A-R	CCCCACAATGTGAGAGATGGCGGATTCCTGAAGGTTGGCC
NotI-SLFN-F	TTATAGCGGCCGCATGAACATCAGTGTTGATTTG
MluI-SLFN12-R	ATCGACGCGTTCAGGTGAGCCTTCGACA
SLFN-I105N-F	GTAACTACTTTCTGAATTTTGTGAAGTCATG
SLFN-I105N-R	TTCAGAAAGTAGTTACCATTCTGCATGAAGTC

Supplementary note

Nauclefine was synthesized starting from Di-*t*-butyloxycarboryl protected 2-ethynyl tryptamine. After Sonogashira coupling with methyl 4-iodonicotinate and subsequent deprotection, we obtained the cascade reaction precursor, of which the amino was engaged into the crucial nucleophilic addition to the alkynyl in Cs_2CO_3/MeOH with excellent regioselectivity (exo-type) followed by concomitant cyclization to generate C ring of nauclefine 3. We also employed the same strategy to accomplish the synthesis of Angustine (14). Prior to the cascade cyclization to afford 20-bromonauclefine (21), the precursor 20B was produced by Sonogashira coupling from 19B and 19C. Finally, the vinyl group was introduced by PPh_3-catalyzed Stille coupling to yield Angustine (14) (scheme 1). For the synthesis of subditine, the C ring of the subditine skeleton was established by referring to Lavilla's method[30]. As shown in scheme 1 in the supplementary note, the condensation of compound 22 with 5-bromo-6-methylnicotinoyl chloride 23 gave 24 accompanied by the isomerization of 1-cyclic olefinic bond, which then underwent 6π electric cyclization at 190 ^{o}C *in vacuo* to afford 17 in 48% yield. Subsequently, we also installed a vinyl group into the pyridine ring by Stille coupling reaction (15), the structure of which was verified by X-ray single crystal diffraction. Further dihydroxylation and oxidation (in one pot) under strict temperature control yielded the target subditine (Supplementary Fig.12).

Oxygen- and moisture-sensitive reactions were carried out under nitrogen atmosphere. Solvents were purified and dried using standard methods. ^{1}H and ^{13}C NMR spectra were recorded on Bruker-500, 400 spectrometers. Chemical shifts for ^{1}H and ^{13}C NMR spectra are reported in ppm (δ) relative to residue protium in the solvent (^{1}H, δ 7.26 ppm for $CDCl_3$; ^{13}C, δ 77.00 ppm for $CDCl_3$; the multiplicities are presented as follows: s = singlet, d = doublet, t = triplet, q = quartet, m = multiplet. High-resolution mass spectra (HRMS) were acquired on a Waters Micromass GCT Premier or Bruker Daltonics Inc. APEXIII 7.0 TESLA FTMS. Mass spectra were acquired on an Agilent 5975C. The full details of the synthesis of nauclefine and compounds 2-12 are available in a previous report. [1]

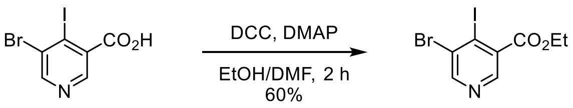

18B **19B**

To the solution of 5-bromo-4-iodonicotinic acid (18B, 300 mg, 0.91 mmol, 1.0 equiv.) in DMF (1.5 mL) was added DMAP (17 mg, 0.14 mmol, 0.15 equiv.), ethanol (0.16 mL, 2.74 mmol, 3.0 equiv.) and DCC (453 mg, 2.19 mmol, 2.4 equiv.) in sequence; after stirring for 2 h at RT, the reaction mixture was filtered through diatomite. The obtained filtrate was washed with brine, dried over anhydrous Na_2SO_4, and then concentrated *in vacuo* to afford the crude product, which was purified by silica gel column chromatography (5~10% ethyl acetate-petroleum ether) to give 19B (196 mg, 60%) as light yellow solid. R_f = 0.59 (30% ethyl acetate-petroleum ether)。 ^{1}H NMR (400 MHz, $CDCl_3$) δ 8.74 (s, 1H), 8.61 (s, 1H), 4.45 (q, *J* = 7.1 Hz, 2H), 1.43 (t, *J* = 7.1 Hz, 3H); ^{13}C NMR (101 MHz, $CDCl_3$) δ 165.0, 152.3, 147.3, 135.4, 131.5, 112.0, 62.6, 14.1 ppm; HRMS (EI, m/z): $[M]^+$ calcd. for $C_8H_7BrINO_2$, 354.8705; found, 354.8706.

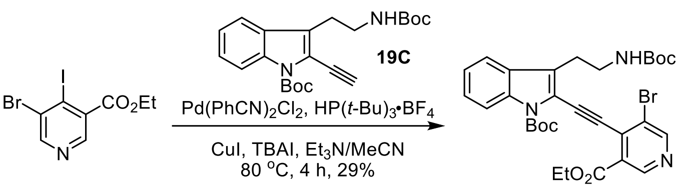

Under N_2 atmosphere, to the mixture of 19C (Prepared according to our previous reported method[1], 54 mg, 0.14 mmol, 1.0 equiv.), ethyl 5-bromo-4-iodonicotinate (19B, 50 mg, 0.14 mmol, 1.0 equiv.), $Pd(PhCN)_2Cl_2$ (6 mg, 0.014 mmol, 0.1 equiv.), CuI (3 mg, 0.014 mmol, 0.1 equiv.), $HP(t\text{-}Bu)_3BF_4$ (8 mg, 0.028 mmol, 0.2 equiv.) and TBAI (155 mg, 0.42 mmol, 3.0 equiv.) was added the solution of Et_3N in acetonitrile (deoxygenated, 1:4, 1.4 mL), followed by stirring at 80 ^{o}C for an additional 4 h. The reaction mixture was filtered through diatomite. The obtained filtrate was concentrated *in vacuo* to afford the crude product, which was purified by silica gel column chromatography (5~30% ethyl acetate-petroleum ether) to give 20B（25 mg, 29%）as a light yellow solid. R_f = 0.58 (30% ethyl acetate-petroleum ether). ^{1}H NMR (400 MHz, $CDCl_3$) δ 9.06 (s, 1H), 8.92 (s, 1H), 8.07 (d, *J* = 8.4 Hz, 1H), 7.66 (d, *J* = 7.8 Hz, 1H), 7.40 (t, *J* = 7.8 Hz, 1H), 7.28 (t, *J* = 7.2 Hz, 1H), 5.12 (s, 1H), 4.45 (q, *J* = 7.1 Hz, 2H), 3.49 (dd, *J* = 13.0, 6.5 Hz, 2H), 3.21 (t, *J* = 6.9 Hz, 2H), 1.67 (s, 9H), 1.41 (t, *J* = 7.1 Hz, 3H),

1.39 (s, 9H); ^{13}C NMR (100 MHz, $CDCl_3$) δ 163.9, 155.9, 154.2, 149.3, 149.0, 136.1, 132.7, 130.0, 128.8, 127.9, 126.7, 124.3, 123.2, 119.8, 117.3, 115.5, 96.8, 92.8, 84.5, 78.9, 62.0, 40.7, 28.3 (3 C), 28.1 (3 C), 25.4, 14.1 ppm; HRMS (ESI, m/z): $[M+Na]^+$ calcd. for $C_{30}H_{34}N_3O_6NaBr$, 634.1529, found 634.1517.

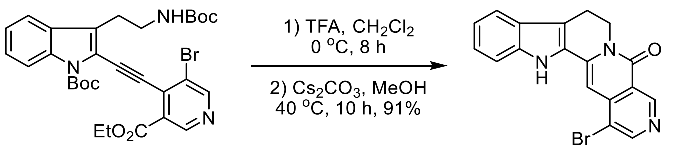

20B **21**

At 0 °C, to the solution of 20B (1.0 equiv.) in DCM (0.3 mL) was added a TFA solution in DCM (0.1 mL, V_{TFA} : V_{DCM} = 1:3), and after stirring for additional 8 h at 0 °C, most of the solvent was evaporated *in vacuo*, diluted with toluene, and then concentrated, which was repeated again. After that, the residue was further evaporated *in vacuo* using an oil pump for 1 h followed by dissolving in methanol (0.7 mL). To the solution, under N_2 atmosphere, Cs_2CO_3 solution in methanol (0.5 M in dry methanol, 0.18 mL, 0.09 mmol, 5.0 equiv.) was added dropwise, followed by stirring at 40 °C for 10 h. The reaction was finally quenched using a saturated NH_4Cl solution, extracted by CH_2Cl_2 (5×10 mL), and then washed by saturated $NaHCO_3$ solution and brine, dried over anhydrous Na_2SO_4. After being concentrated *in vacuo*, the obtained crude residue was purified by silica gel column chromatography (20~40% ethyl acetate- CH_2Cl_2) to afford 21 as a light yellow solid. R_f = 0.50 (5% MeOH - CH_2Cl_2). ^{1}H NMR (300 MHz, DMSO) δ 12.07 (s, 1H), 9.23 (s, 1H), 8.87 (s, 1H), 7.64 (d, J = 7.9 Hz, 1H), 7.47 (d, J = 8.2 Hz, 1H), 7.28 (t, J = 7.4 Hz, 1H), 7.14 (s, 1H), 7.10 (t, J = 7.5 Hz, 1H), 4.40 (t, J = 6.7 Hz, 2H), 3.16 (t, J = 6.7 Hz, 2H); ^{13}C NMR (125 MHz, DMSO) δ ppm; HRMS (EI, m/z): $[M]^+$ calcd. for $C_{18}H_{12}BrN_3O$, 365.0164; found 365.0162.

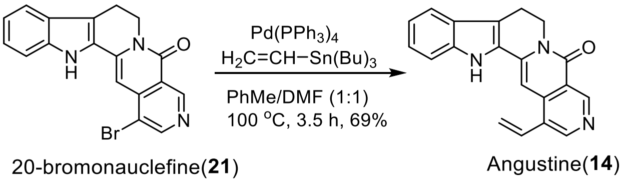

20-bromonauclefine(**21**) Angustine(**14**)

Under N_2 atmosphere, the solution of toluene and DMF (deoxygenated, 15.5 mL) was

injected in to the mixture of 21 (114 mg, 0.31 mmol, 1.0 eq.) and $Pd(PPh_3)_4$ (35.97 mg, 0.031 mmol, 0.1 eq.), after adding tributyl(vinyl)tin (86.4 μL, 0.34 mmol, 1.1 eq.), the resultant solution was then stirred at 100 $_{o}$C for 3 h. After cooling to RT, the reaction solution was filtered through diatomite. The filtrate was diluted with EtOAc (30 ml), and then washed by deionized water (5 mL) and brine (5 mL), dried over anhydrous Na_2SO_4. After being concentrated *in vacuo*, the obtained crude residue was purified by silica gel column chromatography (50% ethyl acetate-CH_2Cl_2) to afford 14（68mg, 69%）as a light yellow solid. R_f = 0.28 (5% MeOH-CH_2Cl_2). ^{1}H NMR (500 MHz, DMSO-*d*$_6$) δ 11.81 (s, 1H), 9.23 (s, 1H), 8.87 (s, 1H), 7.63 (d, *J* = 8.0 Hz, 1H), 7.48 (d, *J* = 8.2 Hz, 1H), 7.31 – 7.19 (m, 3H), 7.10 (t, *J* = 7.5 Hz, 1H), 6.06 (d, *J* = 17.6 Hz, 1H), 5.62 (d, *J* = 10.8 Hz, 1H), 4.40 (t, *J* = 6.7 Hz, 2H), 3.12 (t, *J* = 6.7 Hz, 2H) ppm. ^{13}C NMR (126 MHz, DMSO-*d*$_6$) δ 161.4, 150.0, 148.0, 139.3, 138.8, 137.2, 130.5, 128.1, 127.1, 125.8, 124.9, 120.3, 120.1, 119.5, 119.5, 115.1, 112.3, 94.1, 40.7, 19.5 ppm; HRMS (EI, m/s): [M]$^+$ calcd. for $C_{20}H_{15}N_3O$, 313.1215; found 313.1212.

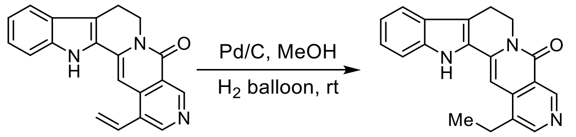

Angustine(**14**) **13**

To a round-bottom flask was added a solution of angustine (8 mg, 0.03 mmol, 1.0 eq.) in 10% CH_2Cl_2/MeOH (3 mL, 0.01M) followed by Pd-C（50% content, 8mg）and 5 drops of AcOH. The atmosphere was fast replaced by nitrogen for three times and the flask was filled with a hydrogen balloon, which was then kept stirring for 4 h at RT. The reaction mixture was finally filtered through diatomite and the obtained filtrate was neutralize by saturated Na_2CO_3 solution. The aqueous phase was extracted by CH_2Cl_2 (4×5mL) and dried over anhydrous Na_2SO_4. After concentrated in vacuo, the obtained crude residue was purified by silica gel column chromatography (60% ethyl acetate-CH_2Cl_2) to afford 13 as a yellow solid（6 mg, 74%）, R_f = 0.27 (5% MeOH/ CH_2Cl_2). ^{1}H NMR (500 MHz, DMSO-*d*$_6$) δ 11.85 (s, 1H), 9.19 (s, 1H), 8.54 (s, 1H), 7.63 (d, *J* = 7.9 Hz, 1H), 7.48 (d, *J* = 8.2 Hz, 1H), 7.27 (t, *J* = 7.1 Hz, 1H), 7.21 (s, 1H), 7.10 (t, *J* = 7.5 Hz, 1H), 4.40 (t, *J* = 6.7 Hz, 2H), 3.12 (t, *J* = 6.7 Hz, 2H), 2.91 (t, *J* = 7.5 Hz, 2H), 1.31 (t, *J* = 7.5 Hz, 3H) ppm. ^{13}C NMR (125 MHz, DMSO-*d*$_6$) δ 161.7, 150.1, 148.9, 140.3, 138.8, 137.0, 132.6, 128.2, 125.8, 124.8, 120.2, 120.1, 119.5, 114.9, 112.3, 94.4, 63.2, 22.8, 19.6, 15.6 ppm; HRMS (ESI, m/s): [M+Na]$^+$ calcd. for $C_{20}H_{17}N_3NaO$, 338.1269; found 328.1243.

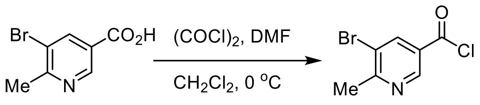

22A **22**

Under N_2 atmosphere, at 0 ^{o}C, to the solution of 5-bromo-6-methylnicotinic acid (22A, 300 mg, 1.4 mmol, 1.0 eq) in dried CH_2Cl_2 (14 ml, 0.1 mol/L) was added one drop of DMF and oxalyl chloride (2.38 ml, 2.0 M, 2.4 eq.) dropwise. After stirring at RT for 90 min, the reaction was evaporated *in vacuo* to afford crude 22, which was used in the next step without any purification.

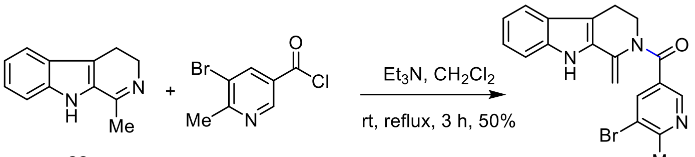

22 **23** **24**

Under N_2 atmosphere, at 0 ^{o}C, to the solution of 23 (250 mg, 1.1 mmol, 1.0 eq.) in CH_2Cl_2 (10 mL) was added Et_3N (6 ml) dropwise, the reaction was then raised to RT and stirred for additional 1 h. After that, 22 (250 mg, 1.34 mmol, 1.2 eq.) was added to the reaction and then stirred at 45 ^{o}C for 2 h, which was then evaporated *in vacuo* and purified by silica gel column chromatography (15% ethyl acetate-petroleum ether) to afford crude 24（230mg, about 50%, decomposed quickly at room temperature）as a yellow solid. R_f = 0.63 (30% ethyl acetate-petroleum ether).

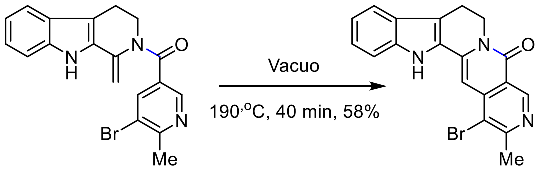

24 **17**

Compound 24 (230 mg, 0.6 mmol, 1.0 eq.) was added to a 50 mL round-bottom flask equipped with a double row tube, after N_2 replacement three times, the flask was set on a 190 ^{o}C oil bath and kept *in vacuo* for 30min. When cooled to RT, the obtained crude solid was purified by silica gel column chromatography (50% ethyl acetate-CH_2Cl_2) to afford 17（113 mg, 58%）as a yellow solid. R_f = 0.65 (10% Methanol-CH_2Cl_2). ^{1}H NMR (500 MHz, Chloroform-*d*) δ 9.40 (s, 1H), 8.67 (s, 1H), 7.65 (d, *J* = 7.9 Hz, 1H), 7.49 (d, *J* = 8.2 Hz, 1H), 7.37 (t, *J* = 7.1 Hz, 1H), 7.23 (t, *J* = 7.5 Hz, 1H), 6.88 (s, 1H), 4.54 (t, *J* = 6.7 Hz, 2H), 3.20 (t, *J* = 6.7 Hz, 2H), 2.83 (s, 3H) ppm. ^{13}C NMR (125 MHz, Chloroform-*d*) δ 161.4, 159.5, 149.3, 141.6, 138.5, 137.4, 127.2, 125.9, 125.5, 121.0, 119.9, 119.1, 117.2, 116.8, 111. 8, 96.2, 40.6, 26.1, 19.8 ppm; HRMS (ESI, m/s): $[M+H]^{+}$ calcd. for $C_{19}H_{15}N_3OBr$, 380.0398; found 380.0393.

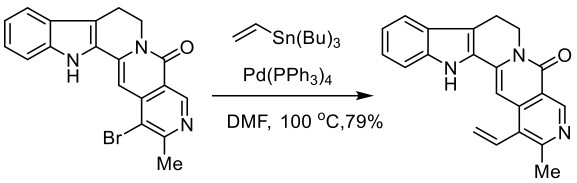

17 **15**

To a 50 mL round-bottom flask was added 17（50 mg, 0.13 mmol, 1.0 eq.）and $Pd(PPh_3)_4$ (15.2 mg, 0.013 mmol, 0.1 eq.), which was replaced with three times with N_2 , followed by injection of DMF (deoxygenated, 13 mL, 0.01 mol/L) and Tributyl(vinyl)tin (38.4 μL, 0.13 mmol). The reaction continued for 3 h at 100 ^{o}C, cooled to RT, and then was filtered through diatomite. The filtrate was diluted with EtOAc (50 mL), washed with deionized water (10 mL) and brine (15 mL), and finally dried over anhydrous Na_2SO_4. After being concentrated *in vacuo*, the obtained crude residue was purified by silica gel column chromatography (50% ethyl acetate-petroleum ether) to afford 15 (34mg, 79%) as yellow solid. R_f = 0.50 (10% MeOH-CH_2Cl_2). ^{1}H NMR (500 MHz, Chloroform-*d*) δ 9.44 (s, 1H), 8.62 (s, 1H), 7.64 (d, *J* = 8.0 Hz, 1H), 7.46 (d, *J* = 8.2 Hz, 1H), 7.34 (t, *J* = 7.7 Hz, 1H), 7.21 (t, *J* = 7.6 Hz, 1H), 6.93 – 6.79 (m, 2H), 5.83 (d, *J* = 11.4 Hz, 1H), 5.54 (d, *J* = 17.9 Hz, 1H), 4.54 (t, *J* = 6.7 Hz, 2H), 3.18 (t, *J* = 6.7 Hz, 2H), 2.66 (s, 3H) ppm. ^{13}C NMR (125 MHz, Chloroform-*d*) δ 162.2, 149.5, 139.8, 138.3, 136.0, 131.8, 127.6, 126.4, 126.1, 125.1, 122.9, 120.8, 119.7, 118.2, 116.0, 111.6, 95.1, 77.2, 40.5, 23.7, 19.8 ppm; HRMS (ESI, m/s): $[M+H]^+$ calcd. for $C_{21}H_{18}N_3O$, 328.1450; found 328.1436.

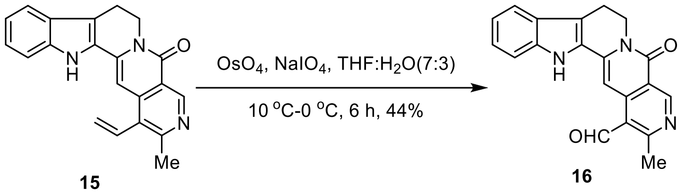

15 **16**

At 10 ^{o}C, to the solution of 15 (90 mg, 0.27 mmol, 1.0 eq.) in THF/H_2O (10 mL, 70% THF in water) was added a solution of OsO_4 in toluene (10 % w/v, 1 ml) in portions over 5 hours. The reaction was then cooled to 0 ^{o}C, followed by adding $NaIO_4$ (587 mg, 2.75 mmol, 10 eq) with stirring for additional 1 h. The reaction was then quenched by saturated NaHS solution, and extracted with EtOAc (5×10 mL). The combined extraction was washed with deionized water (10 mL) and brine (10 mL), and finally dried over anhydrous Na_2SO_4. After being concentrated *in vacuo*, the obtained crude residue was purified by silica gel column chromatography (60% ethyl acetate-CH_2Cl_2) to afford 16（44mg, 44%）as yellow solid. R_f =

0.40 (10% MeOH-CH_2Cl_2). ^{1}H NMR (500 MHz, Chloroform-*d*) δ 10.73 (s, 1H), 9.58 (s, 1H), 8.95 (s, 1H), 8.04 (s, 1H), 7.62 (d, *J* = 7.9 Hz, 1H), 7.47 (d, *J* = 8.1 Hz, 1H), 7.35 (t, *J* = 7.6 Hz, 1H), 7.22 – 7.18 (m, 1H), 4.52 (t, *J* = 6.7 Hz, 2H), 3.19 (t, *J* = 6.8 Hz, 2H), 3.00 (s, 3H) ppm. ^{13}C NMR (125 MHz, Chloroform-*d*) δ 192.6, 165.9, 163.9, 161.6, 155.2, 141.1, 139.3, 138.6, 127.3, 125.8, 125.7, 121.0, 120.3, 119.9, 117.2, 111.9, 94.7, 40.5, 22.7, 19.8 ppm; HRMS (ESI, m/s): $[M+H]^+$ calcd. for $C_{20}H_{16}N_3O_2$, 330.1243; found 330.1232.

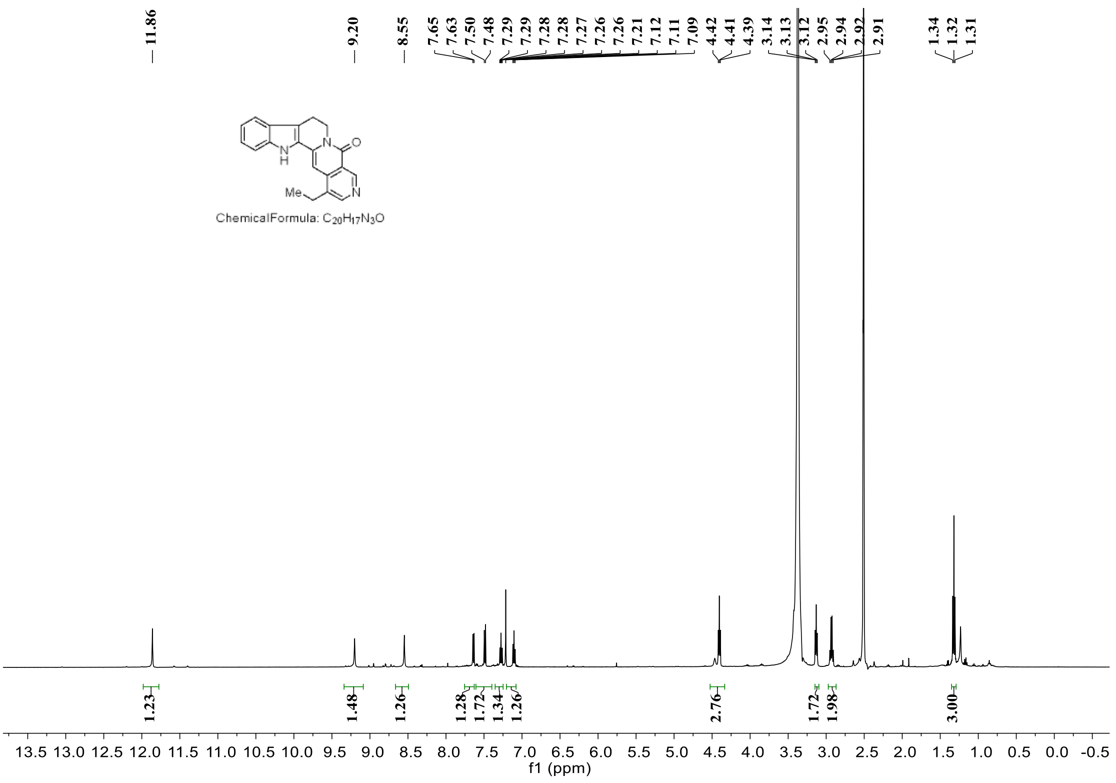

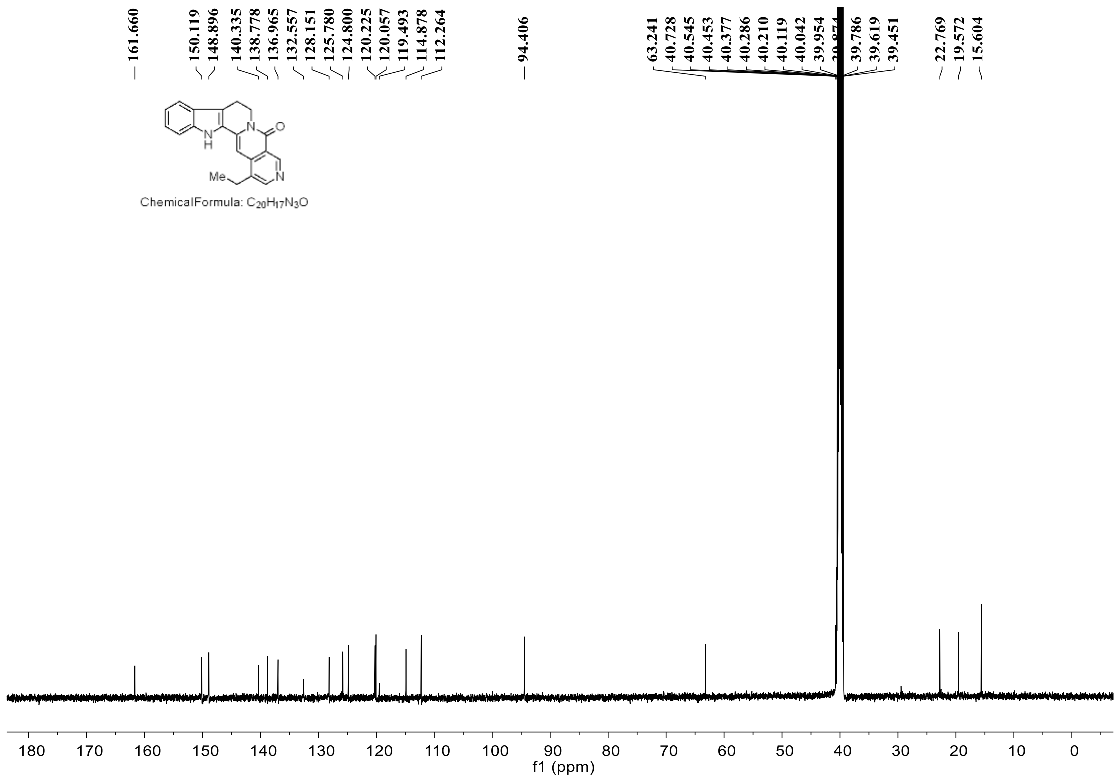
161.660
150.119
148.896
140.335
138.778
136.965
132.557
128.151
125.780
124.800
120.225
120.057
119.493
114.878
112.264
94.406
63.241
40.728
40.545
40.453
40.377
40.286
40.210
40.119
40.042
39.954
39.786
39.619
39.451
22.769
19.572
15.604
ChemicalFormula: $C_{20}H_{17}N_3O$
f1 (ppm)

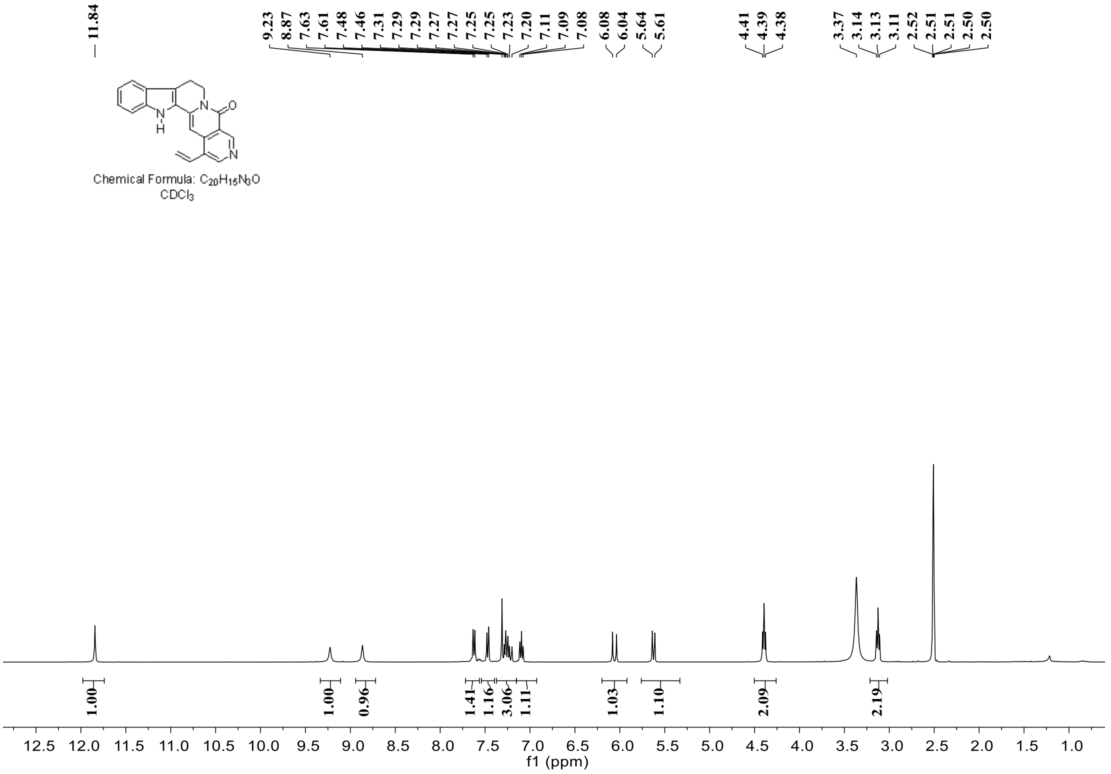
11.84
9.23 8.87 7.63 7.61 7.48 7.46 7.31 7.29 7.29 7.27 7.27 7.25 7.25 7.23 7.20 7.11 7.09 7.08 6.08 6.04 5.64 5.61
4.41 4.39 4.38
3.37 3.14 3.13 3.11 2.52 2.51 2.51 2.50 2.50
Chemical Formula: C20H15N3O
CDCl3
1.00 1.00 0.96 1.41 1.16 3.06 1.11 1.03 1.10 2.09 2.19
f1 (ppm)

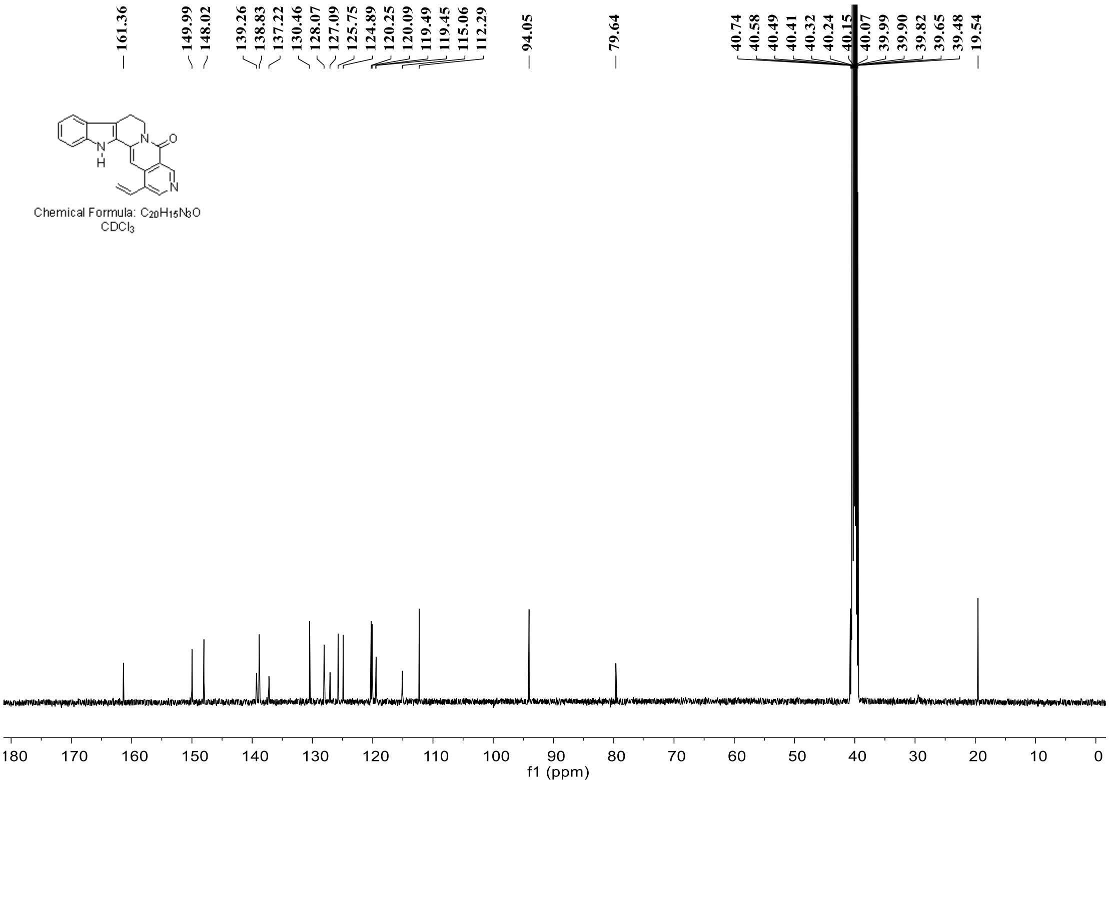
161.36
149.99 148.02
139.26 138.83 137.22 130.46 128.07 127.09 125.75 124.89 120.25 120.09 119.49 119.45 115.06 112.29
94.05
79.64
40.74 40.58 40.49 40.41 40.32 40.24 40.15 40.07 39.99 39.90 39.82 39.65 39.48 19.54
Chemical Formula: C20H15N3O
CDCl3
f1 (ppm)

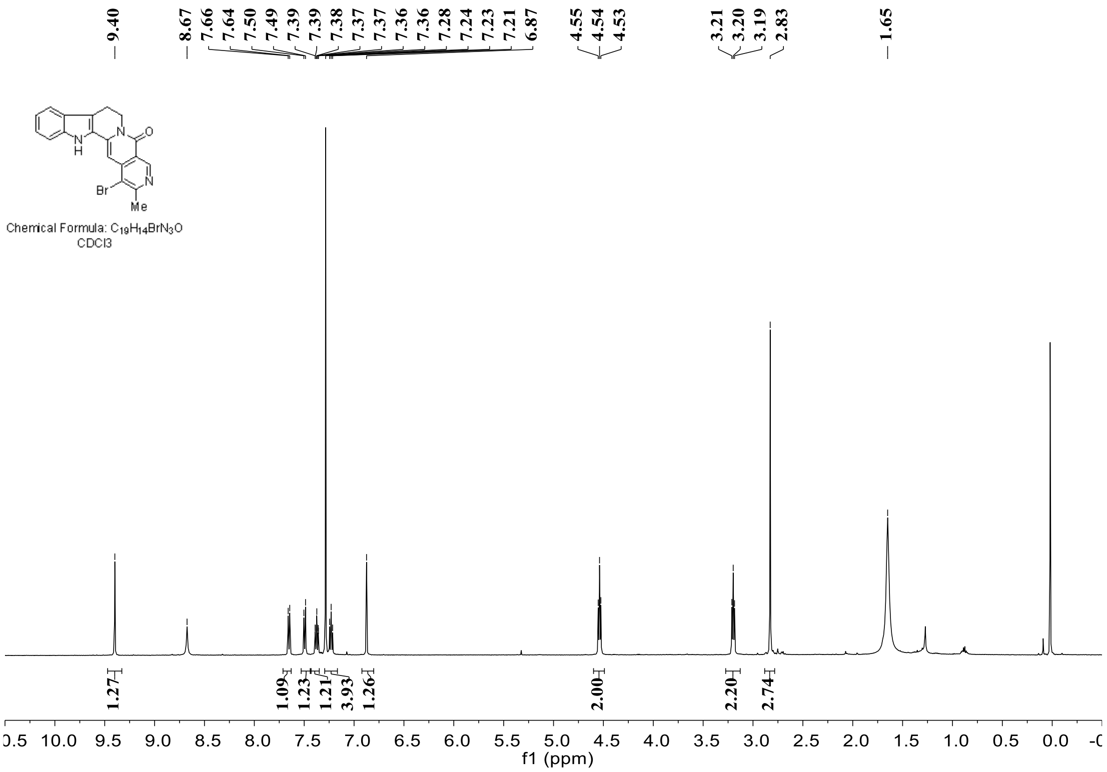
9.40
8.67 7.66 7.64 7.50 7.49 7.39 7.39 7.38 7.37 7.37 7.36 7.36 7.28 7.24 7.23 7.21 6.87
4.55 4.54 4.53
3.21 3.20 3.19 2.83
1.65
Br
Me
N
H
O
Chemical Formula: $C_{19}H_{14}BrN_3O$
CDCl3
1.27 1.09 1.23 1.21 3.93 1.26 2.00 2.20 2.74
f1 (ppm)

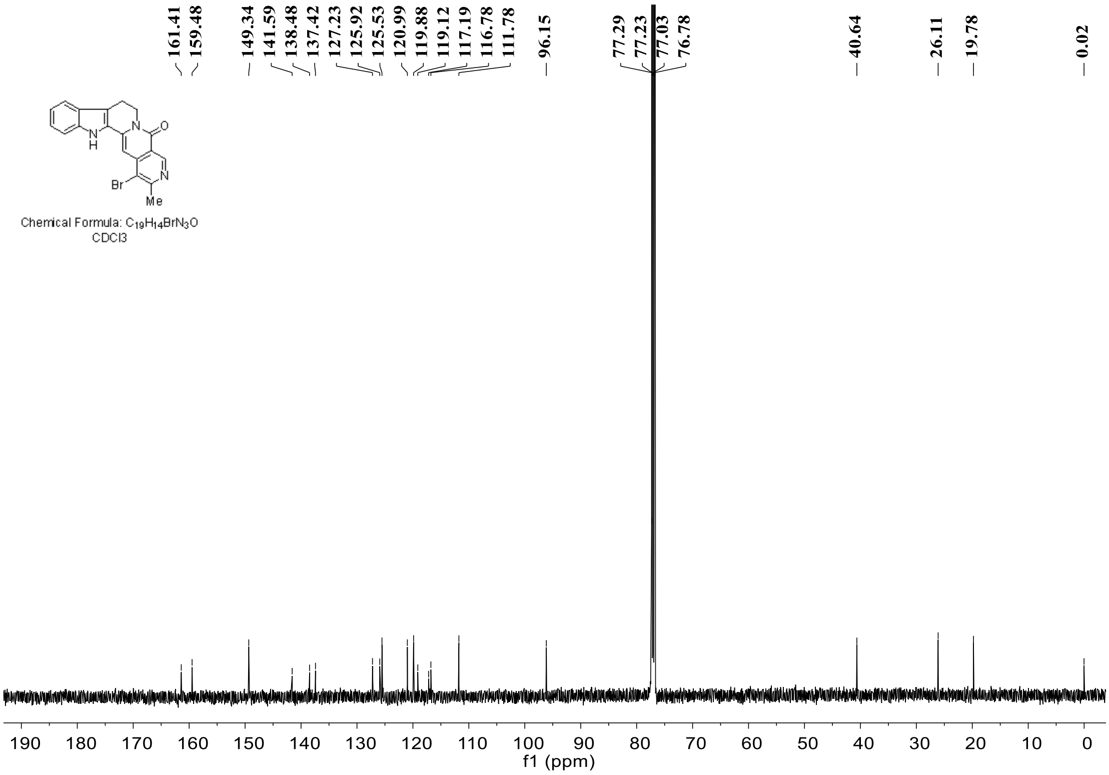
161.41 159.48
149.34 141.59 138.48 137.42 127.23 125.92 125.53 120.99 119.88 119.12 117.19 116.78 111.78
96.15
77.29 77.23 77.03 76.78
40.64
26.11 19.78
0.02
Br
Me
N
H
O
Chemical Formula: $C_{19}H_{14}BrN_3O$
CDCl3
f1 (ppm)

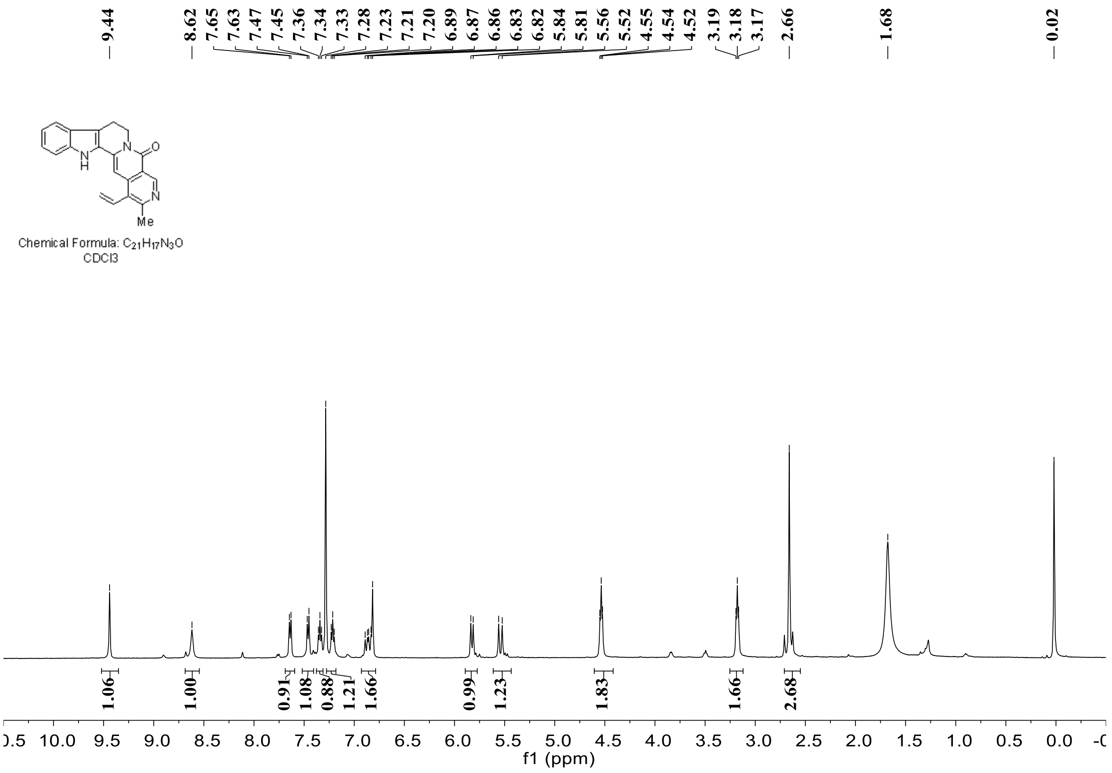
9.44
8.62 7.65 7.63 7.47 7.45 7.36 7.34 7.33 7.28 7.23 7.21 7.20 6.89 6.87 6.86 6.83 6.82 5.84 5.81 5.56 5.52 4.55 4.54 4.52
3.19 3.18 3.17
2.66
1.68
0.02
NH
O
N
Me
Chemical Formula: $C_{21}H_{17}N_3O$
CDCl3
1.06 1.00 0.91 1.08 0.88 1.21 1.66 0.99 1.23 1.83 1.66 2.68
10.0 9.5 9.0 8.5 8.0 7.5 7.0 6.5 6.0 5.5 5.0 4.5 4.0 3.5 3.0 2.5 2.0 1.5 1.0 0.5 0.0
f1 (ppm)

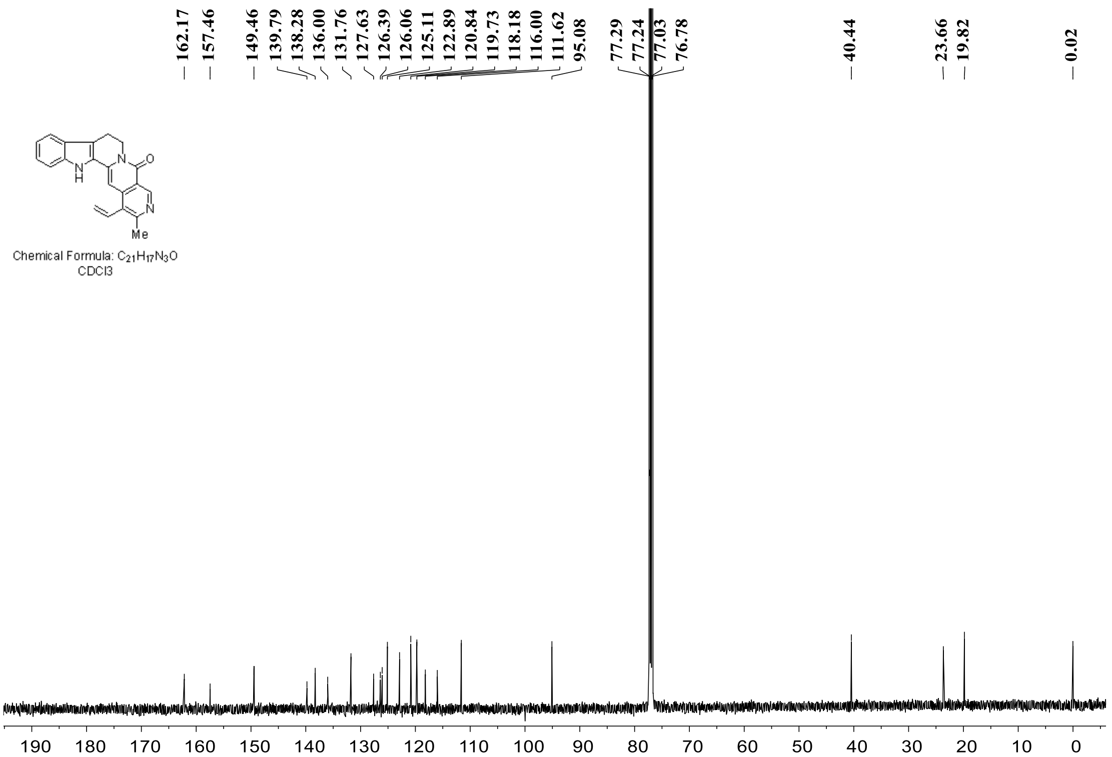
162.17
157.46
149.46 139.79 138.28 136.00 131.76 127.63 126.39 126.06 125.11 122.89 120.84 119.73 118.18 116.00 111.62
95.08
77.29 77.24 77.03 76.78
40.44
23.66 19.82
0.02
NH
O
N
Me
Chemical Formula: $C_{21}H_{17}N_3O$
CDCl3
190 180 170 160 150 140 130 120 110 100 90 80 70 60 50 40 30 20 10 0
f1 (ppm)

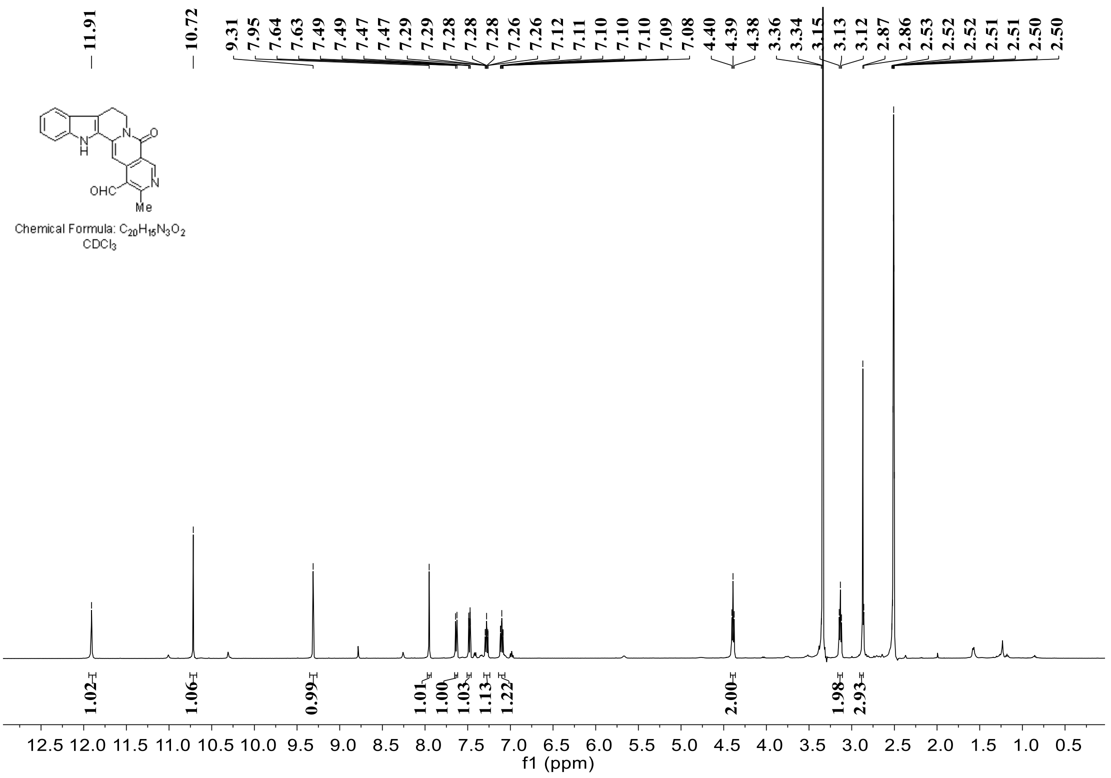

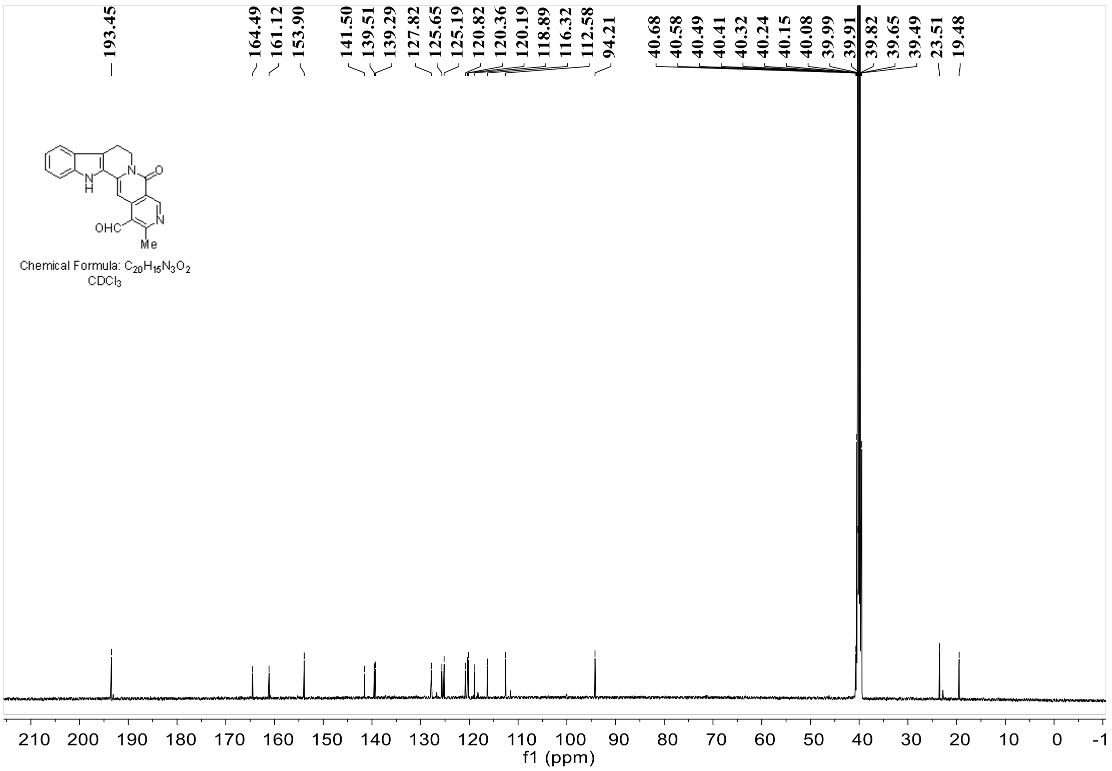

Supplementary Reference

1. K. Li, J. Ou, Shuanhu Gao, Total Synthesis of Camptothecin and Related Natural Products by a Flexible Strategy *Angew. Chem. Int. Ed.* 2016, *55*, 14778.
