# Supplementary material for: An alkaloid initiates phosphodiesterase 3A–schlafen 12 dependent apoptosis without affecting the phosphodiesterase activity
Source: Nat Commun. 2020 Jun 26;11:3236. doi: 10.1038/s41467-020-17052-4 (PMC7319972; doi:10.1038/s41467-020-17052-4)
Supplement: Supplementary file 2 — Reporting Summary [file 41467_2020_17052_MOESM2_ESM.pdf]

nature research

Corresponding author(s): Youwei Ai, Jianzhang Ma, Shuanhu Gao, Xiangbing Qi

Last updated by author(s): Jun 2, 2020

Reporting Summary

Nature Research wishes to improve the reproducibility of the work that we publish. This form provides structure for consistency and transparency in reporting. For further information on Nature Research policies, see our Editorial Policies and the Editorial Policy Checklist.

Statistics

For all statistical analyses, confirm that the following items are present in the figure legend, table legend, main text, or Methods section.

n/a	Confirmed	
☐	☒	The exact sample size (*n*) for each experimental group/condition, given as a discrete number and unit of measurement
☐	☒	A statement on whether measurements were taken from distinct samples or whether the same sample was measured repeatedly
☐	☒	The statistical test(s) used AND whether they are one- or two-sided<br>*Only common tests should be described solely by name; describe more complex techniques in the Methods section.*
☒	☐	A description of all covariates tested
☒	☐	A description of any assumptions or corrections, such as tests of normality and adjustment for multiple comparisons
☒	☐	A full description of the statistical parameters including central tendency (e.g. means) or other basic estimates (e.g. regression coefficient) AND variation (e.g. standard deviation) or associated estimates of uncertainty (e.g. confidence intervals)
☐	☒	For null hypothesis testing, the test statistic (e.g. *F*, *t*, *r*) with confidence intervals, effect sizes, degrees of freedom and *P* value noted<br>*Give P values as exact values whenever suitable.*
☒	☐	For Bayesian analysis, information on the choice of priors and Markov chain Monte Carlo settings
☒	☐	For hierarchical and complex designs, identification of the appropriate level for tests and full reporting of outcomes
☒	☐	Estimates of effect sizes (e.g. Cohen's *d*, Pearson's *r*), indicating how they were calculated

Our web collection on statistics for biologists contains articles on many of the points above.

Software and code

Policy information about availability of computer code

Data collection	Nikon A1 microscope was used for image acquisition; BD FACSAria III was used for flow cytometry; Tecan GENios microplate reader was used for cell viability determination.
Data analysis	NIS-Elements (Nikon) softwares were used for image analyses. GraphPad Prism 8.0.2 was used for for statistical analyses. FlowJo (version 10.4.0) was used for flow cytometer analysis. Agilent MassHunter software (Ver. B.07.00) was used for LC-MS/MS analysis. IVIS Spectrum was used to visualize tumor size.

For manuscripts utilizing custom algorithms or software that are central to the research but not yet described in published literature, software must be made available to editors and reviewers. We strongly encourage code deposition in a community repository (e.g. GitHub). See the Nature Research guidelines for submitting code & software for further information.

Data

Policy information about availability of data

All manuscripts must include a data availability statement. This statement should provide the following information, where applicable:
- Accession codes, unique identifiers, or web links for publicly available datasets
- A list of figures that have associated raw data
- A description of any restrictions on data availability

There are no restrictions on data availability. All of other relevant data are available upon requesting from the lead corresponding author, Y.A.. All raw blots corresponding to the SDS-PAGE gels (Fig.1d, 2e-f, 3a-b, 4b-c, 5a-d, Supplementary Fig. 6b, 6d, 7c, 8a, 8c, 9c-f, 11g) and remaining primary data of interest (Fig.1a, 2c-d, 3b-d, 4a,4d,6b, Supplementary Fig.3, 4, 5, 6a, 6c, 7c, 8b, 9b-d, 10b, 11a-f, 11h) are included as Source Data file.

Field-specific reporting

Please select the one below that is the best fit for your research. If you are not sure, read the appropriate sections before making your selection.

☒ Life sciences ☐ Behavioural & social sciences ☐ Ecological, evolutionary & environmental sciences

For a reference copy of the document with all sections, see nature.com/documents/nr-reporting-summary-flat.pdf

Life sciences study design

All studies must disclose on these points even when the disclosure is negative.

Sample size	The cell viability assays were determined with 3 or 4 wells each sample (detailed n is indicated in the figure legends) to perform statistical testing, within 2 or 3 independent experiments. Micrograph or gels were repeated for 2 to 3 times. Sample size of each group of mice was n=4 or 5.
Data exclusions	No data or samples were excluded from the analysis
Replication	All data (except for mice experiments) shown were obtained from at least 2 biological independent experiments.The mice experiments were performed by at least 4 mice in each group and the conclusion was confirmed in two independent xenograft models. Western blots were repeated for 2 to 3 times.
Randomization	Samples were allocated random. For the animal experiments, we used the same age and sex of mice.
Blinding	The mouse xenograft experiments were done in a blinded fashion.

Reporting for specific materials, systems and methods

We require information from authors about some types of materials, experimental systems and methods used in many studies. Here, indicate whether each material, system or method listed is relevant to your study. If you are not sure if a list item applies to your research, read the appropriate section before selecting a response.

Materials & experimental systems

n/a	Involved in the study	
☐	☒	Antibodies
☐	☒	Eukaryotic cell lines
☒	☐	Palaeontology and archaeology
☐	☒	Animals and other organisms
☒	☐	Human research participants
☒	☐	Clinical data
☒	☐	Dual use research of concern

Methods

n/a	Involved in the study	
☒	☐	ChIP-seq
☐	☒	Flow cytometry
☒	☐	MRI-based neuroimaging

Antibodies

Antibodies used	PDE3A antibody from Bethyl Laboratories (1:1000, Cat# A302-740A); SLFN12 antibody from abcam (1:400, Cat# ab234418); Cleaved-Caspase-3 antibody from Cell Signaling technology (1:1000, Cat# 9661); Caspase-9 antibody from Cell Signaling technology (1:1000, Cat# 9502); PARP antibody from Cell Signaling technology (1:1000, Cat# 9542); Bcl-2 antibody from Cell Signaling technology (1:1000, Cat# 4223); anti-Rabbit-HRP antibody from Sigma-Aldrich (1:5000, Cat# A0545); anti Mouse-HRP antibody from Sigma-Aldrich (1:5000, Cat# A9044); MYC-HRP antibody from MBL (1:1000, M-047-7); Flag-HRP antibody from Sigma-Aldrich (1:10,000, Cat# A8592); Actin-HRP antibody from MBL (1:50,000, Cat# PM053-7); α-Tubulin-HRP antibody from MBL (1:50,000, PM054-7); anti-GAPDH-HRP antibody from MBL (1:50,000, Cat# M171-1).
Validation	All antibodies for western blotting were well-recognized clones in the field and validated by the manufacturers. These antibodies are further validated and routinely used in our lab. Antibodies targeting PDE3A, SLFN12 were validated by KO cells. Antibodies targeting Myc, Actin, Tubulin, GAPDH, Bcl2,CASP9,CASP3,PARP, and Flag were validated by immunoblotting and all the detected-bands were matched with the predicted molecular weight.

Eukaryotic cell lines

Policy information about cell lines

Cell line source(s)	HEK293T and H4 cells were kept in Xiaodong Wang's lab; HeLa, MCF-7; EKVX, SGC-7901, A549 were obtained from the American Type Culture Collection (ATCC).

Authentication	HEK293T and H4 cells were not subjected to cell authentication. The rest cell lines were obtained from commercial source and therefore, not authenticated.
Mycoplasma contamination	All cell lines tested negative for mycoplasma contamination
Commonly misidentified lines (See ICLAC register)	None

Animals and other organisms

Policy information about studies involving animals; ARRIVE guidelines recommended for reporting animal research

Laboratory animals	Female nude mice (Balb/c-nude, 6~7 week). All mice were maintained in animal room with 12 h light/12 h dark cycles, temperature (22–24 °C), humidity (40-60%) at animal facility in National Institute of Biological Sciences, Beijing.
Wild animals	None
Field-collected samples	The study did not involve samples collected from the field.
Ethics oversight	Animal experiments were conducted following the Chinese Ministry of Health national guidelines and performed in accordance with institutional regulations reviewed and approved by the Institutional Animal Care and Use Committee of the National Institute of Biological Sciences, Beijing.

Note that full information on the approval of the study protocol must also be provided in the manuscript.

Flow Cytometry

Plots

Confirm that:

- [x] The axis labels state the marker and fluorochrome used (e.g. CD4-FITC).
- [x] The axis scales are clearly visible. Include numbers along axes only for bottom left plot of group (a 'group' is an analysis of identical markers).
- [x] All plots are contour plots with outliers or pseudocolor plots.
- [x] A numerical value for number of cells or percentage (with statistics) is provided.

Methodology

Sample preparation	Cells were digested by trypsin, washed with PBS, incubate with PI and substrates to stain Annexin V for 10 mins.
Instrument	BD FACSAria III
Software	FlowJo (version 10.4.0, Becton, Dickinson & Company)
Cell population abundance	Analysis 10^6 cells with post-sort populations. cells were gated by FSC and SSC to discard cell adhesion and debris
Gating strategy	Cells are first gated on the basis of their scatter properties. Forward (FSC) and side scatter (SSC) give an idea of the size and granularity of the cells respectively. A naive HeLa cell is used as a known negative control can help to set negative gates and determine real populations.

- [x] Tick this box to confirm that a figure exemplifying the gating strategy is provided in the Supplementary Information.
